# Charge Transport and Carrier Polarity Tuning by Electrolyte Gating in Nickel Benzenehexathiol Coordination Nanosheets

**DOI:** 10.1002/adma.202500164

**Published:** 2025-06-04

**Authors:** Tian Wu, Xinglong Ren, Zhengkang Qu, Ian E. Jacobs, Lu Zhang, Naoya Fukui, Xin Chen, Hiroshi Nishihara, Henning Sirringhaus

**Affiliations:** ^1^ Cavendish Laboratory University of Cambridge 19 J J Thomson Avenue Cambridge CB3 0US UK; ^2^ Research Institute for Science and Technology Tokyo University of Science 2641 Yamazaki Noda‐shi Chiba‐ken 278‐8510 Japan; ^3^ Department of Materials Science & Metallurgy University of Cambridge 27 Charles Babbage Road Cambridge CB3 0FS UK

**Keywords:** coordination nanosheets, conjugated metal organic frameworks, charge transport physics, electrolyte gating, thermoelectric properties

## Abstract

Coordination nanosheets (CONASHs) or conjugated metal organic frameworks (MOFs) with distinctive metal‐organic bonding structures exhibit promise for electronics, sensing, and energy storage. Porous Nickel‐Benzene hexathiol complex (Ni‐BHT) with noteworthy conductivity was first reported a decade ago, and recent synthetic modifications produced non‐porous Ni‐BHT with enhanced conductivity (≈50 S cm^−1^). Here the charge transport physics of such non‐porous Ni‐BHT films are studied with even higher conductivity (≈112 S cm^−1^). In contrast to the thermally activated electrical conductivity, thermoelectric measurements suggest an intrinsic metallic nature of Ni‐BHT. It is shown that it is possible to tune the Fermi level and carrier polarity in Ni‐BHT by electrolyte gating; gating is initially governed by the formation of an interfacial, electric double layer and then evolves into an electrochemical (de)doping process. These findings not only contribute to a deeper understanding of charge transport in CONASHs, but also show that Fermi level tuning is an effective approach for enhancing the thermoelectric performance of CONASHs.

## Graphical abstract

This work reveals that charge transport in nickel benzenehexathiol coordination nanosheets is governed by variable‐range hopping reflecting structural disorder. Electrolyte gating induces a switch in dominant carrier polarity from p‐type to n‐type and leads to an improvement in thermoelectric performance.

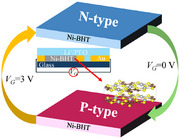

## 1 Introduction

Coordination nanosheets (CONASHs) or conjugated metal organic frameworks (MOFs) have garnered significant attention in the field of two‐dimensional (2D) organic materials.^[^ ^1^, ^2^, ^3^ ^]^ Owing to their unique metal‐organic coordination bonding structures, these materials offer a promising platform for various applications in electronics, sensing, catalysis, and energy storage.^[^ ^4^, ^5^, ^6^, ^7^, ^8^ ^]^ Unlike traditional three‐dimensional (3D) MOFs, CONASHs possess extended conjugation in at least two dimensions, creating highly delocalized π‐electron systems.^[^ ^3^ ^]^ This feature not only gives them excellent electrical conductivity but also opens up avenues for tuning their electronic and optoelectronic properties through molecular design. In 2013, Nishihara's group reported the synthesis and characterization of a nickel benzenehexathiol complex (Ni‐BHT).^[^ ^9^ ^]^ As first reported, Ni‐BHT with a composition of Ni_1.5_BHT had a porous structure with π‐d conjugations along the metal‐ligand bonding, π‐π interactions along the stacking direction and a remarkable conductivity up to 2.8 S cm^−1^ in as‐synthesized films, which is much higher than 3D MOFs.^[^ ^10^ ^]^ Even higher conductivities up to ≈160 S cm^−1^ are achievable upon oxidation of the as‐synthesized films. More recently, Nishihara's group further modified the synthesis process and reported also a non‐porous structure of Ni‐BHT with an elevated ratio of Ni to BHT along with the absence of counterions. A composition of Ni_3_BHT has been reported, similar to that of copper benzenehexathiol, Cu_3_BHT.^[^ ^11^ ^]^ This non‐porous structure of Ni‐BHT was also reported by Dinca's group for its great potential in supercapacitors.^[^ ^12^ ^]^

The conductivity of non‐porous, as‐synthesized Ni‐BHT films, which is around 50 S cm^−1^ at room temperature, surpasses that of its porous structure. This significant one‐order‐of‐magnitude increase is attributed to the establishment of additional transport pathways facilitated by the inserted Ni_6_BHT units within each pore.^[^ ^11^ ^]^ Despite its high conductivity, the non‐porous Ni‐BHT continues to exhibit thermally activated transport.^[^ ^11^ ^]^ This observation implies either transport being limited by defects constituting energetic barriers or the electronic structure of Ni‐BHT being semiconducting (or insulating), which is totally different from Cu_3_BHT which has been reported as metallic material with a similar crystal structure.^[^ ^13^, ^14^ ^]^ There persists a notable gap in our fundamental comprehension of the charge transport mechanisms of intrinsic Ni‐BHT. An in‐depth investigation of the charge transport physics of Ni‐BHT is urgently needed; it would not only provide insights into the intrinsic transport mechanisms but also represents a crucial avenue for exploring strategies to manipulate the electronic properties of this class of materials.

A particular challenge in such CONASHs is the control of the carrier concentration and the position of the Fermi level within the electronic structure through doping. The ability to tune the Fermi level would allow better understanding of the charge transport properties, but may also provide access to exotic transport properties, if the Fermi level could be positioned near a point in the band structure at which topologically insulating behavior has been predicted.^[^ ^15^ ^]^ The theoretically predicted exotic phenomena would only emerge if the carrier concentration could be precisely controlled on the order of 1 charge per unit cell. This level of carrier concentration modulation is unattainable with the commonly used field‐effect gating method. Chemical doping (oxidation/reduction) can significantly alter the carrier concentration, (e.g., the initial report by Nishihara's group reported that Ni‐BHT exhibits its highest conductivity in its oxidized form), but the ability to control the doping level is limited. Electrolyte gating offers a solution by combining the advantages of both approaches, allowing for continuous and reversible tuning of carrier concentration in the range of > 10^13^ cm^−2^ (>10^20^ cm^−3^).^[^ ^16^ ^]^ In particular, to the best of our knowledge few attempts have been made to control doping levels electrically within electrolyte‐gated device architectures, which have allowed efficient control of doping and Fermi level tuning in other classes of functional materials, such as organic semiconductors^[^ ^17^ ^]^ or inorganic oxides.^[^ ^18^ ^]^ A noteworthy study by *Yan*’s group demonstrated the ambipolar behavior of electrochemical transistors (ECTs) based on Cu₃(HHTP)₂ [2,3,6,7,10,11‐hexahydroxytriphenylene (HHTP)], achieving an on/off ratio on the order of 10.^[^ ^19^ ^]^ The operation of Cu₃(HHTP)₂ ECTs benefits from its highly porous crystal structure, which leads to efficient ion diffusion throughout the bulk and high volumetric capacitance. For non‐porous CONASHs, however, it remains elusive whether their properties can be effectively controlled using such electrolyte gating strategy.

Here, we report an in‐depth charge transport study on films of non‐porous Ni‐BHT, that were deposited using an optimized synthetic protocol yielding a nearly twofold higher conductivity up to 112 S/cm than previously reported despite the polycrystalline structure of the films. Temperature‐dependent conductivity measurements reveal an Efros–Shklovskii variable‐range hopping (ES‐VRH) mechanism, which is consistent with temperature‐dependent magnetoresistance (MR). An inconsistency between the polarity of carriers contributing to the Hall effect and the thermoelectric coefficients has been observed, which can be explained by a sign anomaly in the Hall effect in strongly disordered systems.^[^ ^20^, ^21^, ^22^ ^]^ The room temperature carrier mobility is estimated to be ≈2 cm^2^ V^−1^ s^−1^ based on field‐effect transistor measurements, which is probably limited by structural disorder. Based on Nernst effect measurements, we estimate a much higher microscopic mobility of ≈77.6 cm^2^ V^−1^ s^−1^ that could be expected if the structural disorder is fully suppressed. We also demonstrate that an electrolyte‐gated transistor (EGT) architecture based on lithium perchlorate/poly(ethylene oxide) (LiClO_4_/PEO) can be used to tune the carrier polarity of Ni‐BHT, which changes from p‐type to n‐type after gating. This p‐n transition is ascribed to the efficient modulation of charge density on the order of 10^21^ cm^−3^ and tuning of the Fermi level within the band structure, leading to ≈5 times enhancement of thermoelectric power factor.

## 2 Results and Discussion

### 2.1 Structural and Compositional Analysis

The synthetic protocol for our Ni‐BHT films is described in Supporting Information (SI); we used chlorobenzene (CB) for the BHT solution instead of chloromethane in the liquid‐liquid interfacial synthesis method reported in the literature.^[^ ^9^ ^]^ The change of solvent allows for a shorter reaction time (2 hours) for the formation of Ni‐BHT compared with the traditional method. We undertook an extensive optimization of the film growth conditions in the liquid‐liquid interfacial synthesis to achieve optimum electrical conductivity. The optimization of the synthesis process is discussed in Section S1 (Supporting Information) (Figures S1–S7, Supporting Information). The optimized condition is described in Methods.

**Figure 1:**
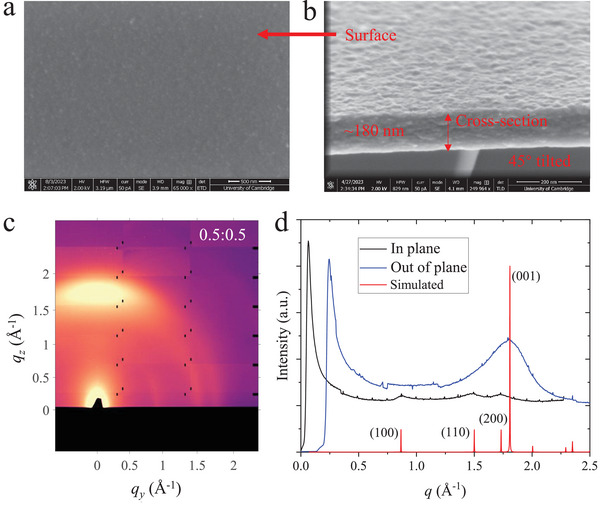
Analysis of thin film microstructure a) SEM image of Ni‐BHT surface; b) Cross‐sectional image of Ni‐BHT; c, d) GIWAXS pattern and integrated 1D linecuts of Ni‐BHT synthesized at a volume ratio of 0.5:0.5 (BHT/CB:NiAc_2_/H_2_O), including a simulated powder diffraction pattern based on the reported Ni‐BHT structure.^[^ ^11^ ^]^ The GIWAXS pattern has a slightly non‐uniform background because it was merged from two images taken at different detector positions to remove the gaps between detector modules.

The surface of Ni‐BHT film is uniform and pin‐hole‐free as indicated by Scanning Electron Microscopy (SEM) (**Figure** **1**a). The flat surface was further confirmed by Atomic Force Microscopy (AFM) images across a 5 µm × 5 µm (Figure S8a, Supporting Information), which exhibited a root‐mean‐square surface roughness of 0.98 nm. This implies a uniform film thickness, which is important for accurate determination of the dimensional parameters needed to interpret the electrical measurements. The uniform and flat film can be further identified by a cross‐sectional SEM image (Figure 1b) from which the thickness of Ni‐BHT film can be extracted to be ≈180 nm. AFM profiles across a cut in the film also indicate a thickness of ≈200 nm (Figure S8b, Supporting Information), which is close to that extracted from the cross‐sectional SEM. Figure 1a and Figure S8a (Supporting Information) also show that the synthesized Ni‐BHT is composed of small grains with a size on the order of 100 nm. From the above morphology characterizations, we conclude that our Ni‐BHT films are smooth, compact and uniform.

Figure S9a (Supporting Information) shows the X‐Ray Photoemission Spectroscopy (XPS) results of synthesized Ni‐BHT which clearly indicates signals from S and Ni with an atomic percentage of 71.74%:28.26%. This ratio (1:2.8) is reasonably close to that expected for non‐porous Ni_3_BHT. However, compositional analysis from XPS has limited accuracy due to uncertainties in the relative sensitivity factor and other instrumental factors^[^ ^23^ ^]^ and the pronounced surface sensitivity of XPS, which means that residual precursors on the surface could also affect the results. The XPS peaks are shifted with respect to the main peak of C 1s at 284.6 eV. The deconvolution of S 2s peaks is shown in Figure S9b (Supporting Information), and peaks at ≈226.0 eV and ≈227.2 eV indicate a mixed 0/‐1 oxidation states of S, which is consistent with reported Ni‐BHT.^[^ ^10^, ^11^ ^]^ The deconvolution of S 2p peaks shown in Figure S9c (Supporting Information) also shows two sets of spin‐orbit split peaks (S 2p_3/2_ and S 2p_1/2_) for the 0 and ‐1 oxidation states, respectively, consistent with the interpretation of the S 2s peak. This implies that some S has shifted to a higher oxidation state due to electron loss during the reaction. Deconvolution of the Ni 2p peaks (Figure S9d, Supporting Information) also reveals two species: The main peaks due to Ni^2+^ appear at ≈854.0 and 871.2 eV, corresponding to the 2p_3/2_ and 2p_1/2_ components. A second species is detected at slightly lower binding energy (852.9 eV for 2p_3/2_ and 870.0 eV for 2p_1/2_), which is attributed to a reduced Ni species with a lower oxidation state, indicating a charge transfer from S to Ni after the formation of Ni‐S bonding, consistent with previous reports.^[^ ^9^, ^11^ ^]^ Satellite peaks were also observed at 860.0 and 875.1 eV.^[^ ^11^, ^12^ ^]^ Ultraviolet X‐Ray Photoelectron (UPS) results of Ni‐BHT (Figure S9a, Supporting Information inset) indicate a finite density of states at the Fermi level, implying that Ni‐BHT should have a metallic nature, similar to the widely reported metallic Cu‐BHT.^[^ ^13^, ^24^ ^]^

Energy‐Dispersive X‐Ray Spectroscopy (EDS) was used for the elemental analysis of the synthesized Ni‐BHT films (Figure S10, Supporting Information). The surface of Ni‐BHT deposited on SiO_x_ substrate exhibits a homogeneous distribution of Ni and S which is consistent with the uniform film morphology as depicted in SEM. The extracted Ni to S weight ratio is around 0.86, which is close to that expected for non‐porous Ni_3_BHT of 0.92, which is considered more accurate than the composition deduced from XPS, as EDS is less surface sensitive than XPS. Here, we have used excess amount of nickel acetate (NiAc_2_) and counterions are absent from the synthesis process which is similar to what has been reported previously.^[^ ^11^ ^]^ Under these conditions, the formation of non‐porous Ni‐BHT is favored, yielding a theoretical Ni‐to‐BHT ratio of 3:1, which closely corresponds to the value estimated via EDS.

Grazing‐Incident Wide Angle X‐Ray Scattering (GIWAXS) was used for structural characterization of Ni‐BHT. As shown in Figure 1c, the 2D diffraction pattern of Ni‐BHT shows a weak diffraction signal with broad, ring‐like features. This observation suggests that the synthesized Ni‐BHT films possess a poly‐ or nano‐crystalline nature without pronounced long‐range ordering. The diminished crystallinity aligns with the nanoparticle morphology observed in SEM and AFM images. The 1D in‐plane and out‐of‐plane linecuts are shown in Figure 1d. A simulated powder diffraction pattern is also shown in Figure 1d based on the reported Ni_3_BHT structure.^[^ ^11^ ^]^ Those peaks aligns well with the GIWAXS data. A hexagonal lattice structure can be constructed with *a* = *b* = 8.38 Å, *c* = 3.48 Å, consistent with the non‐porous structure of Ni‐BHT previously reported, characterized by a Ni/BHT molar ratio of 3.^[^ ^11^ ^]^ This molar ratio aligns closely with the findings from EDS mapping, providing additional confirmation of the non‐porous nature of the synthesized Ni‐BHT. A weak preferred orientation of the grains with the (001) diffraction being observed preferentially in the out‐of‐plane direction is also noticeable. In summary, our film characterization indicates that the structure of our films is fully consistent with literature reports.

### 2.2 Transport Properties

**Figure 2:**
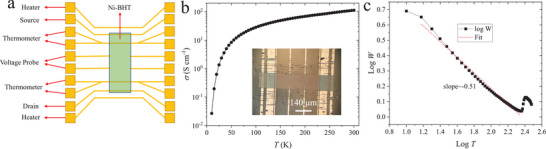
Temperature dependence of electrical conductivity a) Schematic of a multifunctional device; b) Conductivity versus temperature with an inset of real image of the device. The channel length is 420 µm; c) Double logarithmic plot of *W* versus *T* with a slope of ≈‐0.51.

A multifunctional Hall bar device (**Figure** **2**a) was used for the electrical characterization of Ni‐BHT. The films were dimensionally patterned to length (*L*) of 420 µm and width (*W*) of approximately 140 µm by a positioner with a tungsten needle under an optical microscope (Figure 2b inset). To further confirm the thickness (*t*) of the films, a Dektak profilometer was employed, confirming a value of ≈200 nm, which agrees well with the AFM analysis (Figure S11, Supporting Information). Some variations in thickness occurred from sample to sample due to inevitable variations in reaction conditions.

The temperature‐dependent conductivity (*𝜎*) (Figure 2b) exhibits a thermally activated behavior. At 300 K the conductivity can reach 112 S cm^−1^, which is around two‐fold of that reported for non‐porous Ni_3_BHT;^[^ ^11^ ^]^ this high conductivity value may reflect the pin‐hole free and flat morphology of our films (Figure 1a) compared with previous reports. However, at low temperatures the conductivity reduces by more than four orders of magnitude. To analyse the temperature dependence and further understand the charge transport mechanism, a logarithmic derivative method (Zabrodskii analysis) has been used where *W* has been defined as ‐dlog(*R*)/d(log*T*). Here, *R* is the resistance of Ni‐BHT. The results are shown in Figure 2c where log*W* and log*T* have been plotted. The slope is approximately ‐0.5 over a wide temperature range, which means the *𝜎‐T* relation can be written as:

(1) $$\sigma =\sigma_{0}\;\exp\;\left[-{\left(\frac{T_{0}}{T}\right)}^{0.5}\right]$$

Where *𝜎_0_* is the *𝜎* value when *T* is approaching infinity and *T_0_* is a characteristic temperature. This *T^−0.5^* behavior can be interpreted in two ways: One is Efros–Shklovskii variable‐range hopping (ES‐VRH) and the other is inter‐cluster hopping (it is not likely to have 1D Mott VRH in such a 3D‐like nanocrystalline system). In conventional ES‐VRH model, a soft‐gapped density of states near the Fermi energy (*E_F_*) caused by Coulomb interactions would give a ln*σ*∝*T* ^−0.5^ relation.^[^ ^25^, ^26^ ^]^ A similar dependence on *T* can also be derived for thermally assisted tunneling among nanoscale conductive domains within a less conductive matrix.^[^ ^27^ ^]^ This arises from the Coulomb penalty associated with the charging of single carriers. Similar temperature dependence has also been predicted and found in semiconducting polymers.^[^ ^28^, ^29^ ^]^

**Figure 3:**
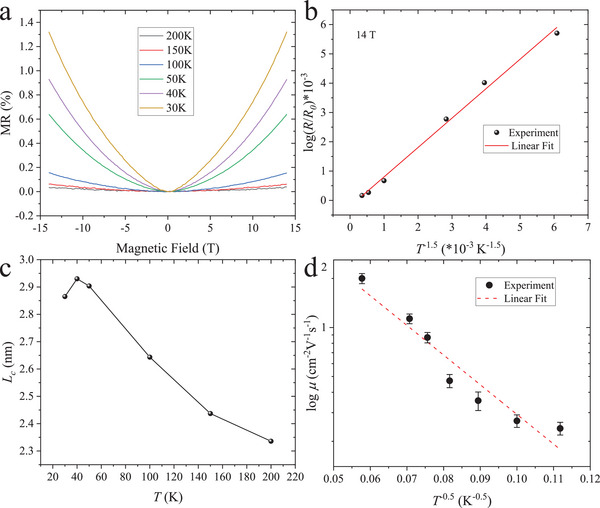
Temperature dependence of magnetoresistance and field‐effect mobility a) Temperature dependent MR; b) Extracted log (*R(14T)/R_0_*) plotted against *T* ^−1.5^; c) Calculated localization length at different temperatures; d) Extracted FET mobilities at different temperatures.

In order to figure out which mechanism is applicable to Ni‐BHT, magnetoresistance (MR) was measured at different temperatures. Here, MR is defined as (*R(B)‐R_0_*)/*R_0_* where *R(B)* is the resistance under magnetic field (*B*) and *R_0_* is the original resistance at *B* = 0. As shown in **Figure** **3**a, the MR of Ni‐BHT remains positive over the whole measured temperature range. When temperature decreases, MR of Ni‐BHT becomes more pronounced. The absence of angular‐dependence in the MR (Figure S12a, Supporting Information) indicates 3D transport, which contradicts at first sight the 2D nature of ideal single‐crystalline Ni‐BHT but can be understood by the nano‐crystalline nature of our samples. The large positive MR at low T can be well fitted with the ES‐VRH model, which, in the weak field limit, can be expressed by:

(2) $$\ln\;\left(\frac{R\left(B\right)}{R_{0}}\right)=t_{1}\;\left[\frac{e^{2}L_{C}^{4}}{{\left(ch\right)}^{2}}\right]{\left(\frac{T}{T_{0}}\right)}^{-1.5}B^{2}$$

Where *t_1_* = 0.0015 for 3D ES‐VRH model, *c* is the speed of light, *h* is Plank constant, *L_c_* is localization length, *e* is elementary charge.^[^ ^25^ ^]^ According to Equation (2), at a given temperature, MR is proportional to *B^2^* in the weak field limit, which agrees well with the experimental data (Figure S12b, Supporting Information). The resistance ratio at 14T and 0T as a function of temperature can also be well fitted with the equation $\ln\left(\frac{R(B)}{R_{0}}\right)\propto T^{-1.5}$, which is depicted in Figure 3b. The consistency between theory and experiment confirms that ES‐VRH dominates charge transport in Ni‐BHT. Moreover, *L_c_* can be extracted from Equation (2) (Figure 3c). *L_c_* remains between 2 to 3 nm, which is relatively stable over the measured temperature range, implying that the increased MR mainly arises from the explicit temperature factor in Equation (2) rather than from the temperature dependence of *L_c_*. In addition, a Coulomb gap of ≈8.46 meV can be extracted, which also remains relatively stable since the localization length shows no significant change with temperature (details in Section S3, Supporting Information).

To further confirm the transport parameters of Ni‐BHT, field‐effect transistors (FETs) were fabricated in a bottom‐gate, bottom‐contact, four‐point‐probe device architecture. The transfer curves of Ni‐BHT FET are noisy (Figure S13a, Supporting Information), probably due to the rough interfacial contact between Ni‐BHT and SiO_2_. However, it is still clear to see that Ni‐BHT exhibits unipolar p‐type transport behavior over the whole temperature range which contradicts the Hall‐effect‐revealed n‐type feature at high temperatures (Figure S14, Supporting Information). The calculated FET mobilities (*µ_FET_*) are shown in Figure 3d. The room temperature *µ_FET_* is ≈2 cm^2^ V^−1^ s^−1^, from which a carrier concentration of ≈3.6 × 10^20^ cm^−3^ can be estimated (Figure S13b, Supporting Information). The repeatability of the mobility data is shown in Table S1 (Supporting Information) where three different devices have been used for the measurement. The average mobility of 1.8 cm^2^ V^−1^ s^−1^ confirms the reliability of FET measurement. The transport parameters extracted from FETs are more insightful than those inferred from the Hall measurements (Figures S14–S16, Supporting Information). Importantly, the Hall effect here is not described by the band theory and the measured Hall signal cannot be interpreted in terms of the carrier density or polarity in disordered Ni‐BHT (for details see Section S3.1 and S3.2, Supporting Information). However, *µ_FET_* is more reliable as it directly measures the change in conductance upon changing the charge density in the channel with the gate voltage. The temperature dependent *µ_FET_* still follows an ES‐VRH law as ln*µ*_*FET*_∝*T* ^−0.5^. The obtained carrier densities at each temperature are shown in Figure S13b (Supporting Information). The change of carrier density with temperature is relatively small, indicating that the Fermi level position, *E_F_*, is relatively independent of temperature, similar to what is expected in a metal.

### 2.3 Thermoelectric Properties

**Figure 4:**
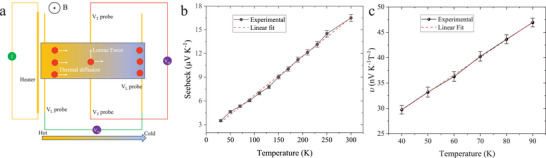
Temperature dependent Seebeck and Nernst coefficients a) Schematic diagram of Seebeck and Nernst effect measurement; red dots: majority carriers; V_T_ probe: transverse voltage probe; V_L_ probe: longitudinal voltage probe; b). Temperature dependent Seebeck coefficient; c. Temperature dependent Nernst coefficient.

The Seebeck coefficient (*S*) is another powerful transport coefficient to identify the polarity of carriers and it also offers crucial insight into the energetic disorder in the density of states and charge carrier transport pathways. The schematic in **Figure** **4**a, illustrates our measurement configuration: An on‐chip heater is utilized to generate an in‐plane temperature gradient, and *S* can be determined by measuring the voltage difference between two electrodes that also serve as resistance thermometers. Additional details are provided in the Supporting Information. The magnitude of *S* is ≈16 µV K^−1^ at room temperature, which is close to that reported for other metallic Ni‐based CONASHs,^[^ ^30^ ^]^ but larger than that of Cu‐BHT.^[^ ^31^ ^]^ *S* decreases with decreasing temperature, but remains positive over the entire measured temperature range, which indicates that Ni‐BHT is p‐type, consistent with the carrier polarity indicated by FET measurements. It is worth noting that *S* is almost proportional to *T* as shown in Figure 4b, a phenomenon widely observed in metallic materials. *S* tends to be less sensitive to structural disorder and grain boundaries than the conductivity;^[^ ^32^ ^]^ the linear relation of *S‐T* indicates that the ordered domains of Ni‐BHT are metallic. The thermally activated conductivity reflects most likely not the intrinsic, metallic nature of Ni‐BHT, but transport barriers associated with defects and grain boundaries. In other words, charge transport in Ni‐BHT is mainly limited by structural disorder.

To further prove the unipolar nature of Ni‐BHT, the temperature dependence of the Nernst coefficient (*υ*) was also measured, as unipolar and ambipolar materials behave differently in the Nernst effect measurements. *υ* is defined in terms of the transverse voltage that is induced by a longitudinal temperature gradient in a vertical magnetic field as shown in Figure 4a:

(3) $$\upsilon =\frac{E_{y}}{B_{z}}\;\frac{1}{\partial_{x}T}$$

Here, *E_y_* is the transverse electric field, *B_z_* is the vertical magnetic field and ∂_x_ *T* is the longitudinal temperature gradient. Substituting *E_y_ = V_xy_/W* and ∂_x_ *T* = *∆T/L* (*V_xy_* is the transverse voltage), the equation can be written as:

(4) $$\upsilon =\frac{L}{W}\;\frac{V_{xy}}{B_{z}}\frac{1}{\Delta T}$$

We restricted our Nernst measurements to the temperature range of 40 – 90 K, around the temperature at which the Hall coefficient exhibits a sign reversal (Figure S15, Supporting Information). The magnitude of the Nernst coefficient in Ni‐BHT is on the order of 40 nV K^−1^T^−1^ at 70 K, which is comparable, but slightly larger than that reported for Cu‐BHT.^[^ ^31^ ^]^ In an ambipolar system, in which holes and electrons coexist, the contributions to *V_xy_* from electrons and holes add‐up in contrast to the Hall effect, for which contributions from electrons and holes cancel. If Ni‐BHT was ambipolar, *υ* should exhibit a peak value at the temperature where the Hall coefficient becomes 0, just like what have been reported in several inorganic ambipolar materials^[^ ^33^, ^34^ ^]^ and in Cu‐BHT.^[^ ^31^ ^]^ However, Figure 4c shows that *υ* is proportional to *T* without any peak found over the temperature range, which further confirms the unipolar nature of Ni‐BHT synthesized. This is further evidence that the sign reversal of the Hall effect cannot be interpreted in terms of a change in the polarity of charge carriers, as discussed above.

The Nernst effect measurements also make it possible to estimate microscopic carrier mobility in disorder‐free Ni‐BHT under the assumption that thermoelectric effects in Ni‐BHT is mainly contributed by the ordered domains. Pioneering work by Behnia^[^ ^35^ ^]^ has suggested that *υ* in metals is set by 2 parameters: *µ* and Fermi temperature *T_F_*. This seemingly simple expression, $\frac{d\upsilon }{dT}∼\left(283\;\mu V\;K^{-1}\right)\frac{\mu }{T_{F}}$, works surprising well for a variety of materials. *T_F_* can be independently extracted from the Seebeck effect measurements. In a free electron model, the hole Seebeck coefficient (*S_h_*) can be expressed by the Mott formula:

(5) $$\;S_{h}=\frac{\pi^{2}k_{B}}{3e}\;\frac{T}{T_{F}}$$

Where *k_B_* is the Boltzmann constant. After obtaining *T_F_* using d*S_h_*/d*T*, the mobility of Ni‐BHT can be estimated to be 77.6 cm^2^ V^−1^ s^−1^. Here we would like to note again that this mobility value (*µ_m__ax_*) is an order‐of‐magnitude estimation for disorder‐free, metallic Ni‐BHT, i.e., without defect/grain boundary transport bottlenecks. Compared with *µ_FET_*, *µ_m__ax_* is nearly 40 times larger. This should be understandable as disorder in Ni‐BHT limits charge transport. Another possible explanation for the large magnitude of *µ_m__ax_* is that the energy dependence of mobility (d*µ*/d*E*) in Ni‐BHT might be much stronger than linear. Behnia's expression assumes that d*µ*/d*E* can be replaced by *µ*/*E* in a first approximation, which works well for good metals but might not be valid for Ni‐BHT. In systems with relatively high charge densities and disordered structures like Ni‐BHT, there might exist phenomena that are strongly energy dependent (e.g., energy filtering by grain boundaries), which could potentially enhance the Nernst effect.

### 2.4 Fermi Level Tuning by Electrolyte‐Gating

**Figure 5:**
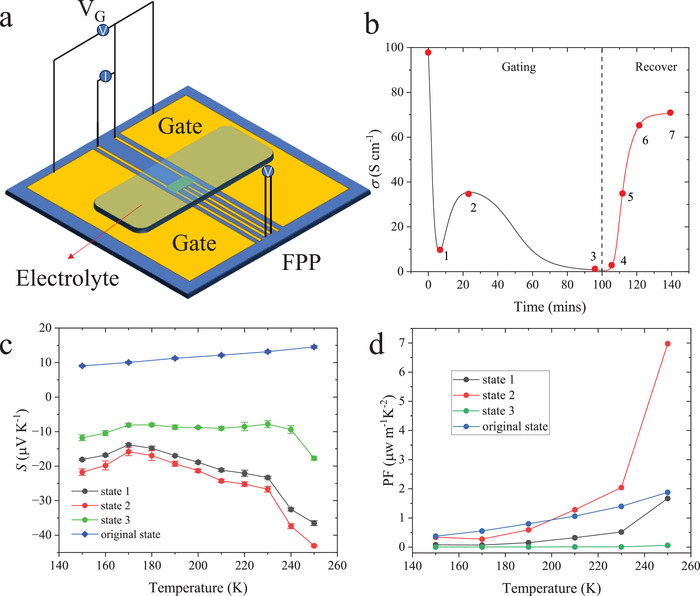
a) Schematic of EGTs; b) Conductivity versus time at *V_G_* = 3 V (Gating) and *V_G_* = ‐1 V (Recover); c) Temperature dependent Seebeck coefficients of state 1–3 and original state; d. Calculated PF values from 150 K to 250 K.

After discussing the charge transport properties of the as‐synthesized Ni‐BHT films, we now turn to a discussion of the use of electrolyte‐gating to modify and tune the carrier concentration and Fermi level position. LiClO_4_/PEO was employed as electrolyte as depicted in **Figure** **5**a. Upon applying a positive gate voltage (*V_G_*), the source‐drain current (*I_sd_*) of Ni‐BHT gradually changes with time (*t*) (Figure 5b). Most interestingly, the current changes in a non‐monotonic faction. While keeping a constant voltage of *V_G_* = 3 V applied, the current and conductivity at first decrease, but after around 300 s, a minimum (state 1) is reached, and beyond this the conductivity rises again. This current increase lasts for around 1000s, before a maximum (state 2) is observed. At longer times, *I_sd_* decreases with time until Ni‐BHT becomes nearly insulating (state 3). This very interesting, non‐monotonic change of *I_sd_* indicates that there are several electronic states of Ni‐BHT involved in this electrolyte‐gating process. After removing *V_G_*, *I_sd_* gradually increases indicating a discharging process for Ni‐BHT. However, as shown in Figure 5b, *I_sd_* cannot recover fully to its original value before charging, implying possible irreversible structural changes and/or electrochemical processes in the Ni‐BHT films upon gating (to be discussed later). Evidence for this is also seen in cyclic voltammetry (CV) measurements as shown in Figure S17 (Supporting Information). The CV scans exhibit no obvious peaks in three subsequent cycles of measurements indicating an absence of reduction/oxidation behavior. This strongly suggests that the observed changes in the conductivity upon gating are not primarily due to electrochemical processes, but capacitive charging, as discussed in more detail below. However, we also observe a decay of current from one cycle to the next (Figure S17a, Supporting Information) indicating some irreversible degradation processes, which is consistent with the incomplete recovery of the conductivity after removing the gate voltage in Figure 5b. It is also worth noting that when applying a higher voltage with the opposite sign in order to inject ClO_4_^−^, the current in the CV scan reduced dramatically, and film was severely damaged (Figure S17b, Supporting Information).

In the following we focus on understanding the different charge states reached upon gating. We selected the individual charge states 1–7 indicated in Figure 5b and measured the transport coefficients *σ* and *S* as a function of temperature. For this we performed the gating at room temperature and upon reaching the desired charge state lowered the temperature to 250 K, before removing the gate voltage. Figure S18 (Supporting Information) shows the temperature‐dependent conductivity measurement of the seven states. The three states obtained during charging (state 1–3) exhibit a similar *ln* *σ*∝*T* ^−0.5^ relation as in the pristine films, indicating an ES‐VRH transport model. The four states during recovery (state 4–7) also show an ES‐VRH transport relation, which indicates that during the entire charging/discharging cycle the films maintain a Coulomb gap in their electronic structure.

To further clarify the polarity of the charge carriers during the charging process, temperature dependent *S* was measured in states 1–3 (Figure 5c). Upon gating, *S* switches sign from the p‐type behavior observed in the pristine films to n‐type behavior. In charge states 1 and 2, *S* exhibits a relatively large negative value (≈−40 µV K^−1^) compared with the pristine films. This change from p‐type to strong n‐type indicates a significant alternation of carrier densities of electrons and holes. The surprising rise of Seebeck coefficient along with a relatively small decrease in conductivity in state 2 leads to a significant enhancement of the thermoelectric power factor (PF) defined as *S* ^2^ *𝜎*. The calculated temperature dependent PF from original state to state 2 is shown in Figure 5d. State 2 exhibits an almost 5‐fold higher *PF* compared with the pristine state. Table S2 (Supporting Information) summarizes the thermoelectric performance of three different devices from state 0 to 2, demonstrating the repeatability of the improved thermoelectric performance upon electrolyte gating. After state 2, the absolute value of *S* decreases while the conductivity decreases, which might be due to some irreversible processes happening in the film. It is also unexpected that all the recovery states show a positive *S* and similar temperature dependence with the original sample as shown in Figure S19 (Supporting Information). Interestingly, if we limit the gating time and stop before reaching state 2, the gating process becomes reversible, and the initial conductivity can be recovered. The n‐type transport behavior can be most cleanly observed when a positive *V_G_* is being applied without inducing irreversible degradation.

**Figure 6:**
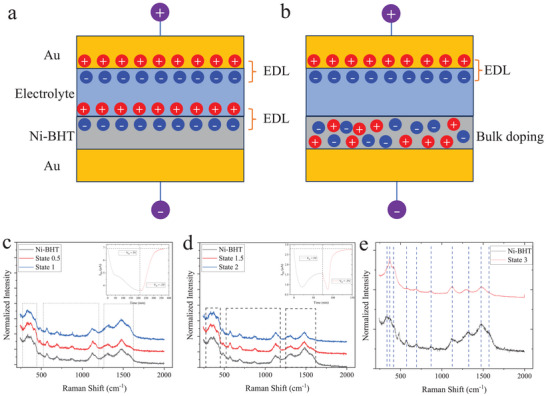
Illustrations of a) EDLTs and b) ECTs; Raman spectra of c) From original state to state 1, inset: time dependent *I_SD_* of an EGT device that only gated to state 1 and its recovery process; d) From original state to state 2, inset: time dependent *I_SD_* of an EGT device that only gated to state 2 and its recovery process; e) From original state to state 3.

In EGTs, there are two mechanisms that can change the polarity of charge carriers. The first mechanism is shown in **Figure** **6**a where two electrical double layers (EDLs) have formed at a given *V_G_*, one at the electrolyte/gate interface and the other one at the Ni‐BHT/electrolyte interface. For a p‐type pristine Ni‐BHT film, the application of a positive *V_G_* would first deplete holes throughout the bulk of the film. Once holes have been depleted, electrons will accumulate in the EDL on the surface, which gradually changes the carrier polarity in the material. This charging mechanism is purely capacitive and does not involve structural changes or electrochemical processes in the bulk, as ions do not penetrate the material. An alternative mechanism shown in Figure 6b involves ions entering the film and inducing bulk doping. This is likely to result in structural changes of the film. A thickness‐dependent impedance measurement was done to characterize whether the charging process is dominated by a 2D EDL or a 3D charging process. As shown in Figure S20 (Supporting Information), the capacitance values for Ni‐BHT films with different thicknesses fluctuate around tens of µF cm^−2^ and there is no correlation between capacitance and thickness. It is evident that this charging process of Ni‐BHT is more like a 2D EDL process.

To further confirm the mechanism, we performed operando Raman spectroscopy on the Ni‐BHT films during charging. No significant changes in the Raman spectrum, that could indicate structural changes due to bulk electrochemical doping, were observed between the Raman spectrum of the pristine state, state 1 and state 2, nor in any intermediate states (defined as state 0.5 and state 1.5 (Figure 6c,d). This reversible process can also be proven by almost full recovery of *I_SD_* when only gated to state 1 or state 2 which is shown in Figure 6c,d insets. State 3 on the other hand exhibits a clear change in the Raman spectrum, indicating structural changes. There are pronounced changes in the relative intensities of several peaks, most prominently in the range of 1000–1500 cm^−1^. There are also shifts in the position of the peaks at 568.65 cm^−1^, 691.67 cm^−1^, 1323.27 cm^−1^. We have not yet attempted to understand the nature of these electrochemical processes and structural changes present in state 3. Our main interest here is to prove that at the beginning of charging process (in state 0 to 2), the charging process is capacitive and the change of *I_SD_* is driven by the formation of an EDL on the surface of the film. The change of polarity is ascribed to the depletion of holes and injection of electrons. After state 2, electrochemical doping of the bulk occurs leading to some irreversible structural changes in the bulk of the films. This process leads to some damage of Ni‐BHT which is indicated by the incomplete recovery of the conductivity. Additional evidence for structural changes is given by XPS measurements on samples gated to state 0 and state 3, as shown in Figure S21 (Supporting Information). A clear shift to lower binding energy of the Ni 2p and S 2p peaks has been observed, suggesting the reduction of Ni‐BHT complex (Figure S21a,b, Supporting Information). This is consistent with electron injection into Ni‐BHT under a positive gate voltage. Most importantly, a clear Li 1s peak has been observed only at state 3 as shown in Figure S21c (Supporting Information), indicating the bulk intercalation of Li ions and structural changes at state 3. UPS measurements show a clear reduction of the density of states at the Fermi level in state 3 which is consistent with the reduced electrical conductivity (Figure S21d, Supporting Information).

The injected number of charges (*N*) can be obtained by integration of the gate current (*I_g_*) with time based on $N=\frac{\int_{0}^{t}I_{g}dt}{e}$. We have compared the time for which Ni‐BHT will reach state 1, in which it becomes electron dominated, for three different samples (Figure S22a—c, Supporting Information). The different samples take different times to reach state 1. This is understandable as the *I_g_* of each sample is not exactly the same due to inevitable difference when preparing the sample (Ni‐BHT dimensions, ion gel thickness, etc.). The corresponding integrated charge densities (*n* = *N/V* where *V* is the volume of each sample) are shown in Table S3 (Supporting Information). The injected charge densities for Ni‐BHT to reach state 1, however, show similar values for the three samples. It takes approximately ≈2 × 10^21^ electrons per cm^−3^ to turn the pristine, p‐type Ni‐BHT to an n‐type material in state 1. Considering that the hole density of the pristine sample is on the order of ≈3.6×10^20^ cm^−3^ (as estimated from FET measurements), it is reasonable to have 10^21^ cm^−3^ electrons injected in order to turn Ni‐BHT n‐type. To note, this number should be overestimated as we cannot fully eliminate the contribution from parasitic current (e.g., time‐dependent leakage current). The error might be on the order of 10%, as suggested by our control experiment using an empty device with only Au electrodes (Figure S22d, Supporting Information), but it should not significantly affect the conclusion.

To explain the observed reversible changes in transport properties upon electrolyte gating into states 1 and 2, we invoke recent calculations of the band structure of the structurally closely related non‐porous Cu‐BHT. Although the 3D bulk band structure calculation of non‐porous Ni‐BHT has not been reported yet, existing experimental studies have suggested that the band structure of Ni‐BHT should be similar to Cu‐BHT.^[^ ^11^ ^]^ Cu‐BHT exhibits a band structure with multiple bands crossing the Fermi level, some with electron character and some with hole character.^[^ ^36^ ^]^ Depending on whether the Fermi level lies in a region of the density of states, which is dominated by bands of either electron or hole character, it would be expected that both the magnitude could be varied and the sign of the Seebeck coefficient flipped by tuning of the Fermi level. This is what we have demonstrated here: By tuning the charge density of Ni‐BHT through electrostatic gating, we have been able to switch the sign and magnitude of the Seebeck coefficient from p‐type to n‐type in a reversible manner; in our as‐synthesized film the Fermi level evidently lies in the region of the density of states, that is dominated by bands of hole character. Upon positive gating we shift the Fermi level to a position, where the conduction channel at the interface becomes dominated by electron bands. This ability to tune the Fermi level within the band structure results in an improved power factor, highlighting the potential of electrolyte gating for optimizing the thermoelectric performance of CONASHs.

## 3 Conclusion

In this paper, we have studied the transport behavior of films of non‐porous Ni‐BHT. An ES‐VRH transport model has been confirmed by temperature dependent conductivity and MR measurements. Ni‐BHT should exhibit a metallic nature, but disorder induced by its low crystallinity limits charge transport and makes it an insulator. A sign anomaly has been observed in the Hall effect, which makes it difficult to interpret the Hall effect measurements in this hopping transport regime. FET measurements and Seebeck measurements provide evidence for predominantly p‐type transport in the as‐synthesized films. The FET mobility of approximately ≈2 cm^2^ V^−1^ s^−1^ is limited by disorder, while an estimate of the carrier mobility in the ordered domains from a combination of Seebeck and Nernst effect measurements provide a much higher upper‐limit value of ≈77.6 cm^2^ V^−1^ s^−1^. Finally, we have demonstrated that EGTs based on LiClO_4_/PEO electrolyte can be used to tune the carrier polarity of Ni‐BHT from p‐type to n‐type and enhance the thermoelectric performance significantly. This is attributed to electrolyte gating being able to tune the Fermi level to a point in the band structure, in which transport near the Fermi level is dominated by electron states in the density of states; this allows reaching reasonably high absolute values of the Seebeck coefficient > 40 µV K^−1^. The initial charging process underpinning the Fermi level tuning occurs by the formation of an EDL on the surface of the film; only upon prolonged gating is there evidence for electrochemical doping and Li incorporation into the bulk of the film. Our study demonstrates that electrolyte gating is an effective method for tuning the electronic properties of CONASHs and for enhancing their thermoelectric performance.

## 4 Experimental Section

### Materials

Nickle acetate tetrahydrate (NiAc_2_.4H_2_O) was purchased from Sigma Aldrich with 99.99% trace metals basis. Benzenehexathiol (BHT) was synthesized according to a previous publication.^[^ ^37^ ^]^ Chlorobenzene (CB, assay >99.9%, water <0.0025%, residue <0.0001%) were purchased from Romil CO. LTD.

### Synthesis

Solution preparation: CB and distilled water were degassed by a continuous flow of nitrogen gas (N₂). CB was degassed for 1 hour, while the water was degassed overnight. Following degassing, both solvents were promptly transferred to the glovebox. Next, 3 mg of BHT was dissolved in 10 ml of CB within a 20 ml vial, and the solution was stirred at 45 °C overnight in the N_2_‐filled glovebox. NiAc₂·4H₂O was dissolved in water at a concentration of 10 mM.

Synthesis: In a 20 ml vial, 6.5 ml of CB was added, followed by the addition of 0.5 ml of BHT/CB solution using a glass syringe with a 0.45 µm PTFE filter. Next, 6.5 ml of water was gently added to the diluted BHT/CB solution using a plastic syringe with a 0.45 µm Nylon filter. The vial was then placed on a hotplate at 45 °C for 10 minutes. Afterward, 0.5 ml of NiAc₂/water solution was slowly and evenly added around the neck of the vial using a plastic syringe with a 0.45 µm Nylon filter, keeping the syringe tip close to the vial wall. The vial remained on the hotplate at 45 °C for 2 hours, during which a dark film gradually appeared within a few minutes.

After the reaction, the vial was opened, and 3 ml of the aqueous phase was removed using a glass pipette. An additional 6.5 ml of water was then added slowly and evenly to the aqueous phase by a plastic syringe with a 0.45 µm Nylon filter, positioning the syringe close to the vial wall to distribute the water around the neck. Approximately 6 ml of water was then removed. This washing procedure was repeated five times.

Next, a substrate, held upside down with the electrode surface facing downward, was carefully lowered by tweezers through the water and water‐CF interface to the bottom of the vial. While holding the tweezers and substrate steady, the water was fully removed using a glass pipette. Once the interface disappeared, the tweezers were slowly lifted from the solution, and the substrate was gently placed on a tissue. After approximately 10 minutes, the substrate with the film on top was dry and was then transferred to a CF solvent for washing for 10 minutes. The substrate was removed and placed on a tissue to dry completely.

### Multifunctional Device Fabrication

The devices were fabricated on 0.7 mm thick Corning 1737F glass substrates via photolithography. First, glass substrates were cleaned by sonication in a sequence of water, acetone and isopropanol (IPA) (5 min each), and then treated by O_2_ plasma for 10 min. After spin‐coating photoresist and photolithography, substrates were transferred into a high‐vacuum evaporator and Cr/Au (3/20 nm) electrodes were deposited by thermal evaporation. The substrates were then left in N‐methyl‐2‐pyrrolidone (NMP) overnight. After lift‐off, all substrates were cleaned by IPA and dried by N_2_ flow. The substates were treated by O_2_ plasma for 10 min before Ni‐BHT film transfer. After transferring the Ni‐BHT film onto the device surface, the film on the undesired region of the device was first removed by tapes and then transferred to a microscope working station for fine patterning.

### Structural and Spectroscopic Characterizations

GIWAXS was performed in Beamline I07 of Diamond Light Source. SEM and EDS were taken by a FEI Helios SEM/FIB. Theta‐2theta X‐ray diffraction was measured by a Bruker D8 Advance powder X‐ray diffractometer. XPS and UPS were measured by a Thermo Scientific Escalab 250xi X‐ray photoemission spectrometer. XPS composition analysis and peak fitting were performed using CasaXPS software. The atomic percentage of an element (*X_*i*_*) was given by:

(6) $$\;X_{i}=100\times \frac{A_{i}}{\sum_{i=1}^{m}A_{i}}$$

where m was the total number of detected elements, and *A_*i*_* was given by *A_*i*_ = I_*i*_ / [T(E) × R_*i*_]*. Here, *I_*i*_* represents the peak intensity, *R_*i*_* was the relative sensitivity factor, and *T(E)* accounts for the instrument's transmission function and escape depth corrections. The parameters embedded within the CasaXPS software were applied for these calculations. More details can be found in the CasaXPS manual.

A Veeco Dimension 3100 atomic force microscope was utilized, operating in tapping mode, to assess the surface morphology and thickness of optimized Ni‐BHT film. Raman spectroscopy (HORIBA Scientific T64000) was measured by 532 nm laser, with 60 s acquisition time and 5 repeated cycles.

### Magnetotransport Measurements

Resistivity, Hall effect and Magnetoresistance (MR) were measured using a Quantum Design Physical Property Measurement System (PPMS) DynaCool system with a DC resistivity puck. The device was stuck on the puck with double‐side copper tape and bonded with Al wires. All the measurements were taken using a constant excitation current (1–50 µA) with a magnetic field step of 0.35 T from ‐14 T to 14 T. More details were described in Section S3 (Supporting Information).

### Calculation of Electrical Conductivities and FET Mobilities

Electrical conductivity was measured based on a four‐point‐probe method (FPP). A voltage sweep (usually from −0.1 V to 0.1 V) was applied between source/drain electrodes while the current (*I_sd_*) flowing through the channel was recorded. The FPP voltage difference (*V_FPP_*) was measured by the two voltage probes in the channel as shown in Figure 2a. The in‐plane dimension of the sample was measured by an optical microscope (distance between voltage probes: *l*, width: *w*) and the thickness (*D*) by a Dektak profilometer. The conductivity was given by:

(7) $$\sigma =\frac{l}{\mathrm{wD}}\;\frac{dI_{\mathrm{sd}}}{dV_{\mathrm{FPP}}}$$

For the resistance (*R*) measured by PPMS, conductivity can be obtained once the dimensions were known:

(8) $$\sigma =\frac{l}{\mathrm{wD}}\;\frac{1}{R}$$

For the FET measurement, a constant source‐drain voltage was applied while the gate voltage (*V_G_*) was swept from ‐50 V to 50 V. Source‐drain current (*I_sd_*) and FPP voltage difference (*V_FPP_*) were recorded. A linear region FET mobility calculation has been applied here:

(9) $$\mu =\frac{l}{wC_{i}}\;d\left(\frac{I_{sd}}{V_{FPP}}\right)/dV_{G}$$

where *C_*i*_* = 11.5 nF cm^−2^, referring to the specific capacitance of 300 nm SiO_2_.

### Thermoelectric Measurements

Seebeck coefficient was measured were performed in a six‐probe closed‐cycle cryogenic probe station (Lake Shore Cryotronics Inc., CRX‐4K) with Keithley 2612B dual‐channel source‐meter units (SMUs) and Keithley 2182 nanovoltmeter based on a previously reported on‐chip geometry. The measurement was taken under high vacuum (<10^−6^ mbar). Seebeck coefficient measurement was based on a previously reported method:^[^ ^38^, ^39^ ^]^

Initially, a current was given to the heater of the device to instigate the generation of a temperature differential (*∆T*). Following a stabilization period of 15 seconds, the thermal voltage (*∆V_xx_*) was detected using a nanovoltmeter. Subsequently, 11 discrete levels of heater power were employed during experimental measurements, revealing a linear correlation between thermal voltage and heater power. The entire procedure was fully controlled by a bespoke JAVA‐based software application.

The slope of the thermal voltage with respect to heater power, denoted as *∆V_xx_*/*∆P*, signifies the longitudinal voltage induced by the temperature gradient. Concurrently, to ascertain the temperature differential between the hot (proximal to the heater) and cold (distal from the heater) side thermometers, resistances of these two thermometers were measured within the same range of heater power, utilizing identical increments. The gradients of thermometer resistance concerning heater power, denoted as *(∆R_hot_)/∆P* and *(∆R_cold_)/∆P*, provide insights into the temperature resistance coefficients of the hot and cold‐side thermometers, represented as *(∆R_hot_)/∆T* and *(∆R_cold_)/∆T*, respectively. These coefficients were deduced from the temperature‐dependent resistance measurements of the thermometers. Seebeck coefficient was then given by:

(10) $$S=\frac{\frac{\Delta V_{xx}}{\Delta P}}{\frac{\frac{\Delta R_{hot}}{\Delta P}}{\frac{\Delta R_{hot}}{\Delta T}}-\frac{\frac{\Delta R_{cold}}{\Delta P}}{\frac{\Delta R_{cold}}{\Delta T}}}=\frac{\Delta V_{xx}}{\Delta T}$$

The Nernst coefficient was measured in the PPMS chamber but using two external Keithley instruments. The temperature was fully controlled by PPMS. A Keithley 2612B SMU was used to supply the heater power and a Keithley 2182 nanovoltmeter was used to measure the transverse thermal voltage. Temperature gradient was determined from temperature resistance coefficients of thermometers at the hot and cold sides of film. The transverse thermal voltage was measured at magnetic fields of −14 T, 0 T, 14 T respectively at the same heater power range.

### Fabrication and Characterization of EGTs

A multifunctional device with patterned Ni‐BHT on top was placed on a tissue. Electrodes of the device (area without Ni‐BHT film) were covered with taps. Three drops of electrolyte solution were dropped cast on top of the device. After drying, the tapes were removed. In this way, the area of electrodes can be minimized in contact with electrolyte.

Charging and discharging of Ni‐BHT EGTs were performed by a probe station in a N_2_‐filled glovebox with a hot plate as the sample holder. Voltage and current were given and recorded by a Keithley 2612B SMU. For low temperature measurements, Ni‐BHT with electrolytes were gated to the desired state by a Keithley 2612B SMU at room temperature. Once it approaches the desired state, the temperature was decreased to lower temperatures (250 K for Seebeck and 190 K for magnetotransport) which would eliminate any ion conduction. Magnetotransport and thermoelectric measurements were performed as described above.

## Conflict of Interest

The authors declare no conflict of interest.

## Supporting information



Supporting Information

## Data Availability

The data that support the findings of this study are available from the corresponding author upon reasonable request.
