# Supplementary material for: Charge Transport and Carrier Polarity Tuning by Electrolyte Gating in Nickel Benzenehexathiol Coordination Nanosheets
Source: Adv Mater. 2025 Jun 4;37(32):2500164. doi: 10.1002/adma.202500164 (PMC12355453; doi:10.1002/adma.202500164)
Supplement: Supplementary file 1 — Supporting Information [file ADMA-37-2500164-s001.docx]

**Charge Transport and Carrier Polarity Tuning by Electrolyte Gating in Nickel Benzenehexathiol Coordination Nanosheets**

Supporting Information

Tian Wu^†1^, Xinglong Ren^†*1^, Zhengkang Qu^1^, Ian E. Jacobs^1^, Lu Zhang^1^, Naoya Fukui^2^, Xin Chen^3^, Hiroshi Nishihara^2^, Henning Sirringhaus^*1^

1. Cavendish Laboratory, University of Cambridge, UK.

2. Research Institute for Science and Technology, Tokyo University of Science, Japan

3. Department of Materials Science & Metallurgy, University of Cambridge, UK.

^†^: These authors contributed equally to this work.

^*^: Corresponding authors: [xr216@cam.ac.uk](mailto:xr216@cam.ac.uk); [hs220@cam.ac.uk.](mailto:hs220@cam.ac.uk)

**Section 1 Modification of Synthesis Process**

We undertook an extensive film optimization process of the key growth parameters in the liquid-liquid interfacial synthesis in order to maximize crystallinity and the electrical conductivity.

**1.1. Precursor Ratios**

We have modified the Ni-BHT film quality by adjusting the precursor ratios of NiAc_2_ and BHT solutions. The amount of NiAc_2_/H_2_O solution added is fixed at 0.5 ml while BHT/CB solution is adjusted from 0.3 ml to 1.2 ml. **From Figure S1, Figure 1a**, it is clear that Ni/BHT synthesized with a ratio of 0.5 ml : 0.5 ml exhibit the clearest GIWAXS diffraction pattern. The in-plane and out-of-plane peak intensities are weaker in other ratios as shown in **Figure S2**. Higher ratio of BHT seems to be better for crystallinity but too much will also deteriorate the ordering.

**
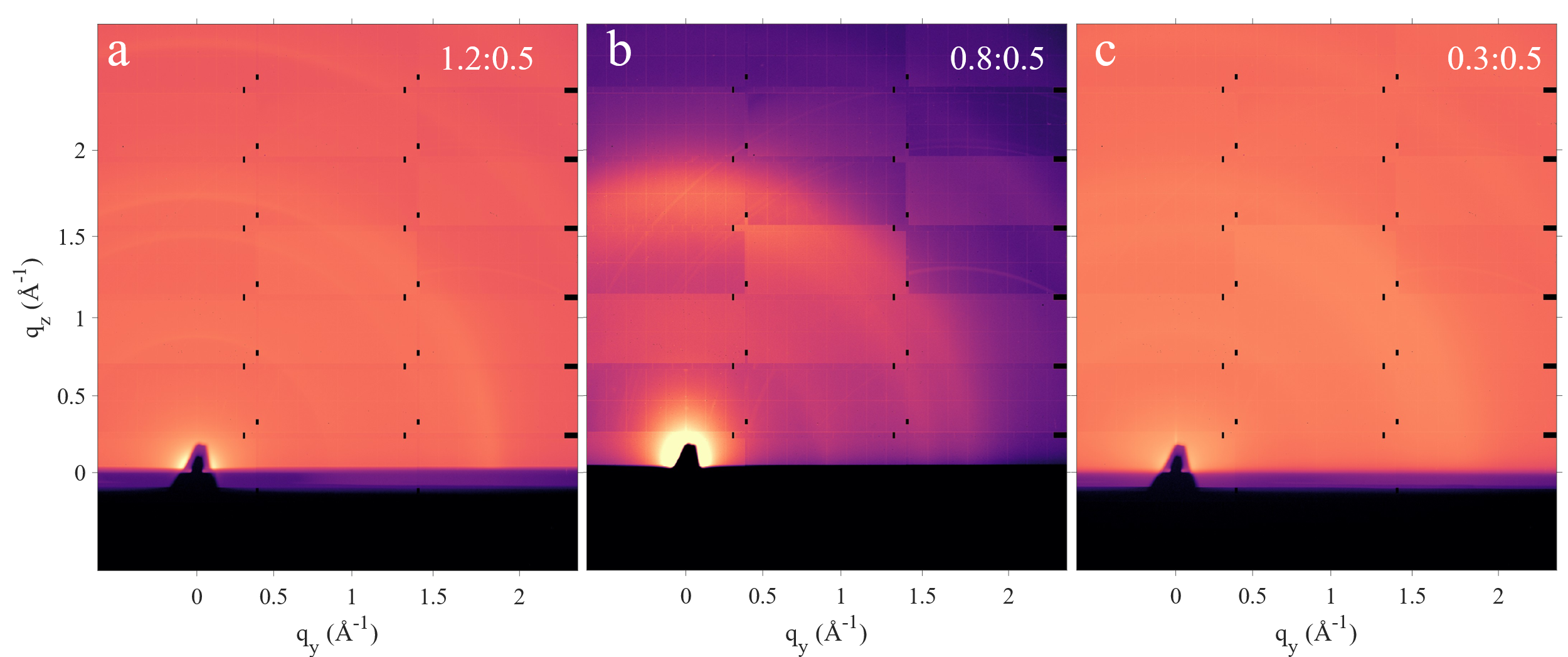
**

**Figure S1** GIWAXS patterns of Ni-BHT synthesized at different precursor (NiAc₂ : BHT) ratios: a. 1.2:0.5; b. 0.8:0.5; c.0.3:0.5.


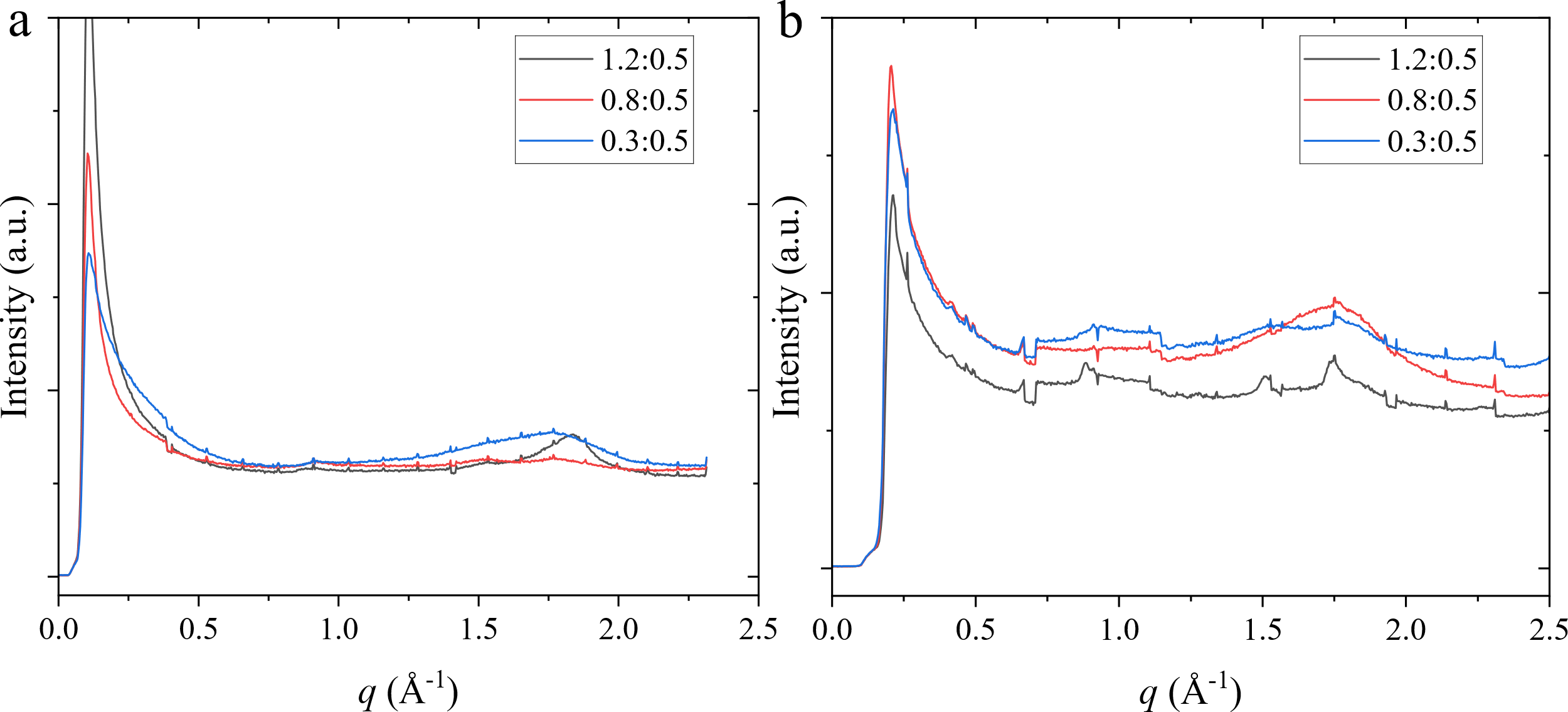


**Figure S2.** a. In-plane and b. out-of-plane linecut of the GIWAXS pattern of Ni-BHT synthesized at different precursor ratios (NiAc₂ : BHT).

However, from the perspective of electrical conductivity, we can see from **Figure S3** that even though ratio of 0.5 : 0.5 (see main text) and 0.8 : 0.5 have better crystallinity, the conductivities of other ratios are still comparable. This indicated that the crystallinity of the films does not strongly impact the conductivity, which suggests that the conductivity is mainly governed by the disordered regions in the films.


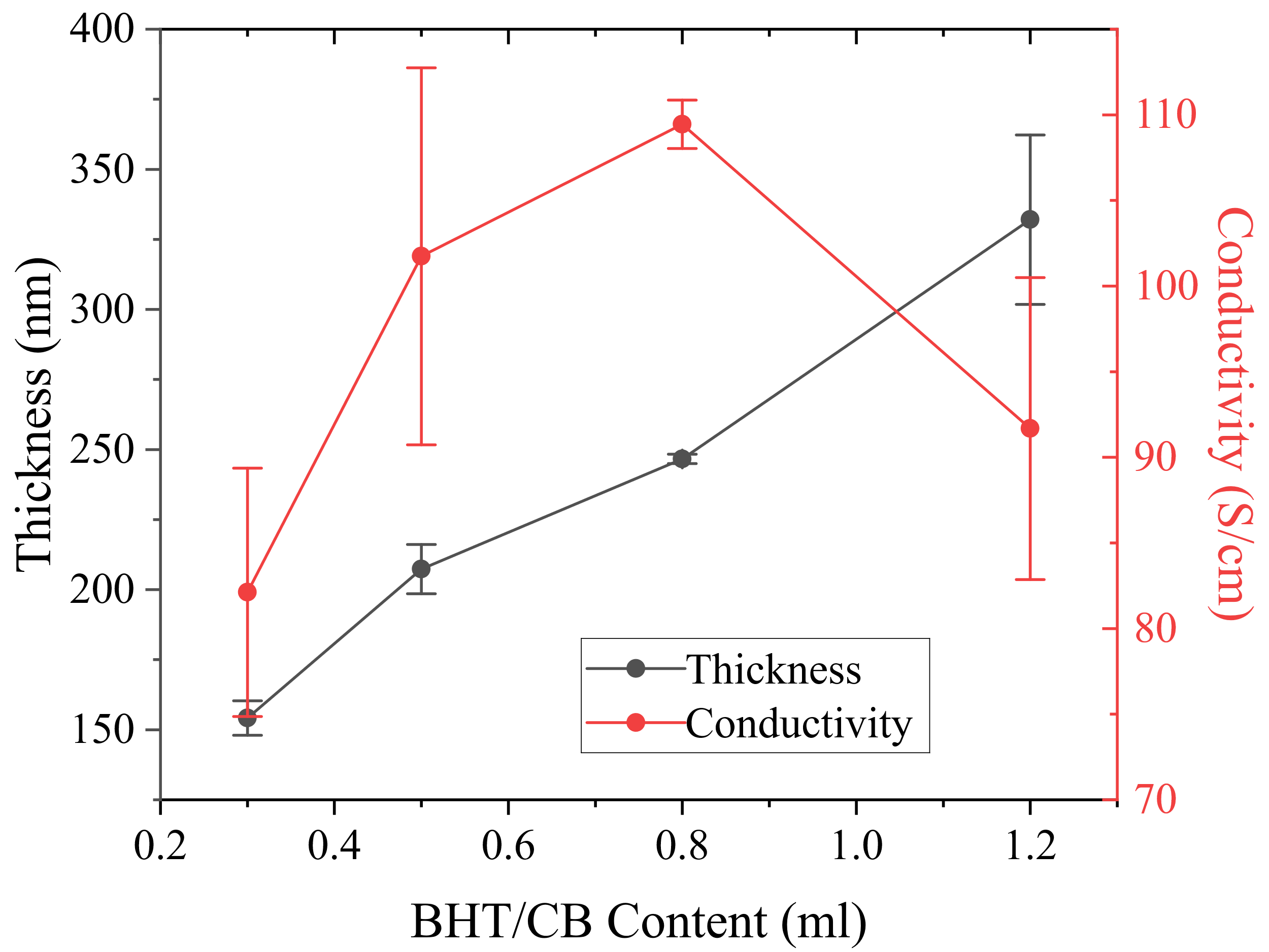


**Figure S3** Thickness and Conductivities of Ni-BHT films synthesized with different amount of BHT/CB solution. The NiAc_2_ added is fixed at 0.5 ml. The data of each ratio is calculated from three different devices.

The reduced conductivity observed for the 1.2:0.5 precursor ratio is attributed to the large thickness of the Ni-BHT film, which exceeds 300 nm. Thicker films tend to be more rigid, and brittle compared to thinner ones. As shown in **Figure S4a**, the film thickness approaches 375 nm, with visible cracks forming along the substrate. These cracks are likely to contribute to the decrease in conductivity. Moreover, such thick films are unsuitable for precise patterning, as illustrated in **Figure S4a**, where the film edges exhibit a zigzag shape, leading to inaccuracies in size measurements. However, it is important to note that the conductivity values in **Figure S3** represent average measurements. Crack-free films with a 1.2:0.5 precursor ratio have been observed, exhibiting a higher conductivity close to 100 S cm^-1^. This suggests that, in the absence of cracks, the conductivity of Ni-BHT films synthesized at this ratio can be comparable to that of films produced with a 0.5:0.5 ratio.


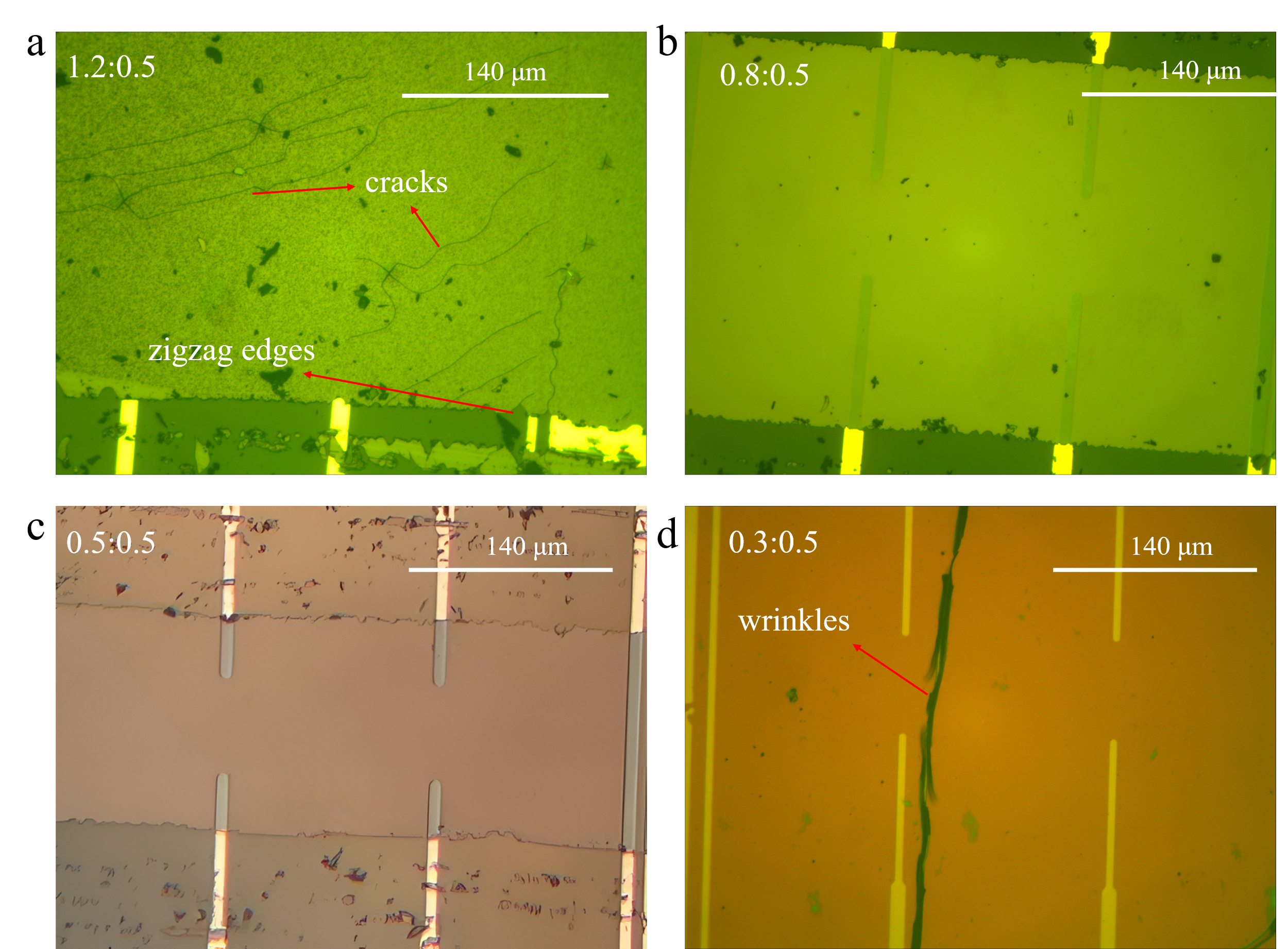


**Figure S4** Optical images of Ni-BHT films synthesized with ratio of a. 1.2:0.5 (thickness ~ 375 nm); b. 0.8:0.5 (thickness ~ 250 nm); c. 0.5:0.5 (thickness ~ 200 nm); d. 0.3:0.5 (thickness ~ 135 nm).

The Ni-BHT films synthesized at a ratio of 0.8:0.5 (**Figure S4b**) and 0.5:0.5 (**Figure S4c**) are more suitable for device fabrication as no cracks have been observed. However, at a 0.3:0.5 ratio, the thickness can be below 150 nm. These thin films also present morphological challenges unsuitable for device fabrication, as shown in **Figure S4d**. Wrinkles commonly appear in thin Ni-BHT films, which not only hinder high conductivity but also compromise the accuracy of conductivity measurements. This “wrinkle” feature was rarely observed in 0.8:0.5 and 0.5:0.5 samples as shown in **Figure S4b** and **S4c**.

**1.2. Thickness**

There are two ways to control the thickness of Ni-BHT film. One way is to adjust concentrations of precursor solutions, and another way is to control the reaction time. We have explored these two methods respectively based on the best ratio in the previous section (0.5:0.5).

**1.2.1 Reaction Time**

The effect of reaction time on the synthesis of Ni-BHT films was investigated over a range of 20 minutes to 4 hours. **Figure S5** presents the correlation between film thickness and electrical conductivity as a function of reaction duration. Notably, when the reaction time is limited to 20 minutes, the resulting Ni-BHT film exhibits a thickness of approximately 70 nm. As the reaction time increases, the film thickness continues to grow; however, the rate of thickness increases gradually decelerates. This trend can be attributed to the formation of the Ni-BHT film at the interface, which acts as a diffusion barrier, thereby limiting the reaction between the two precursors. This phenomenon was also observed in other CONASHs synthesized with this method.^[1]^ Nevertheless, the Ni-BHT film continues to grow and reaches a relatively stable growing rate beyond 1 hour of reaction time.

From an electrical performance perspective, the conductivity of Ni-BHT films approaches its maximum value when the thickness reaches approximately 175 nm. This indicates that a reaction time of 1 hour is sufficient to achieve an optimal Ni-BHT film. However, to ensure the formation of a high-quality and well-developed film, a reaction time of 2 hours was selected for the synthesis process.


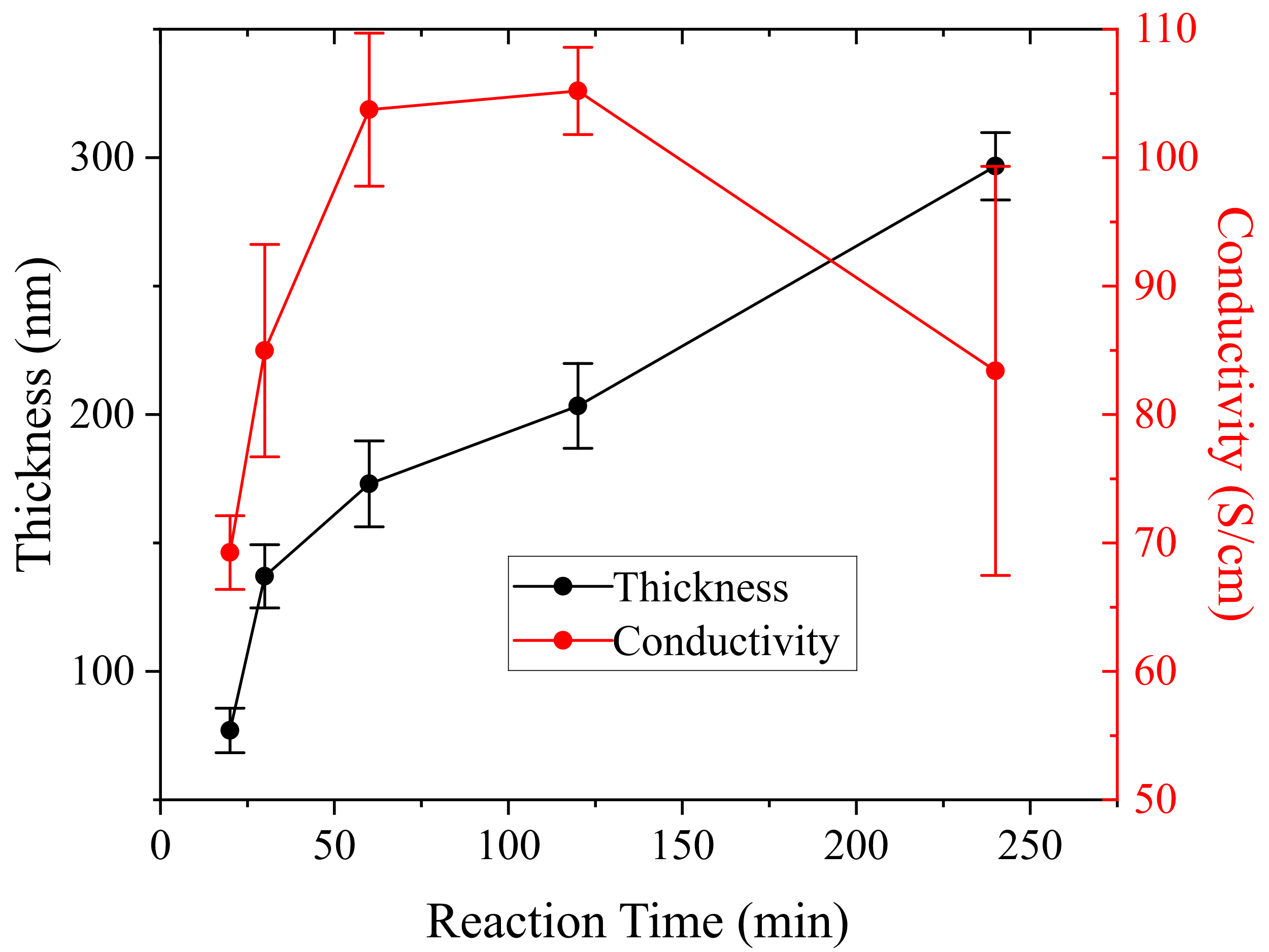


**Figure S5** Thickness and Conductivities of Ni-BHT films synthesized with different reaction time. The NiAc_2_ to BHT ratio is kept with 0.5:0.5. The data of each reaction time is calculated from three different devices.

The thickness requirement for Transmission Electron Microscopy (TEM) analysis is less than 100 nm. We performed TEM measurements on a Ni-BHT film synthesized with a reaction time of 20 minutes, as shown in **Figure S6**. However, no well-ordered domains were observed in the selected area. Several factors may contribute to this. First, Ni-BHT exhibits very low crystallinity, making it difficult to identify an ordered domain with a preferred orientation. Additionally, since the reaction time was limited to 20 minutes, the crystallinity is expected to be even lower compared to that of a film synthesized with 2 hours, which aligns with the reduced conductivity observed in **Figure S5**. Furthermore, our failure to detect crystalline domains of Ni-BHT in TEM may also be attributed to the limited sampling area (on the scale of hundreds of nm²), whereas GIWAXS probes a much larger region (on the scale of cm²).

**Figure S6** a, b. TEM images of Ni-BHT film synthesized with a reaction time of 30 min; c. EDS results of Ni-BHT from the same area shown in **Figure S6a** are consistent with the expected composition of Ni-BHT. No obvious ordered arrays have been observed indicate a near-amorphous structure of Ni-BHT at the beginning of reaction.

**1.2.2 Concentration**

Variations in precursor concentration also leads to differences in film thickness. Here, we define precursor concentration based on the NiAc₂ solution concentration, as it is known (10 mM) and the two precursor solutions are mixed in a 1:1 ratio. Since the total solution volume is fixed at 7 mL, a concentration of 0.714 mM is obtained (using a precursor ratio of 0.5:0.5 as an example). Various concentrations were tested for the synthesis process, ranging from 0.428 mM (precursor ratio of 0.3:0.3) to 1.429 mM (precursor ratio of 1.0:1.0). Lowering the concentration further results in extremely thin Ni-BHT films that are too fragile to withstand the washing and transfer process, making subsequent electrical measurements impossible.

Thickness and conductivity measurements for different concentrations are presented in **Figure S7**. The film synthesized at 0.428 mM has a thickness of approximately 125 nm, similar to that observed for a reaction time of 30 minutes. The conductivity for 0.428 mM is comparable to that of the optimal concentration (0.714 mM). The "wrinkle" issue seen in **Figure S4b** also appears in the 0.428 mM film, which makes the standard deviation significantly larger. For films synthesized at higher precursor concentrations, the thickness can exceed 300 nm. However, such thick films also suffer from cracking shown in **Figure S4a**. As expected, the average conductivity of these thicker films is lower than that of films with the optimal thickness (~200 nm).


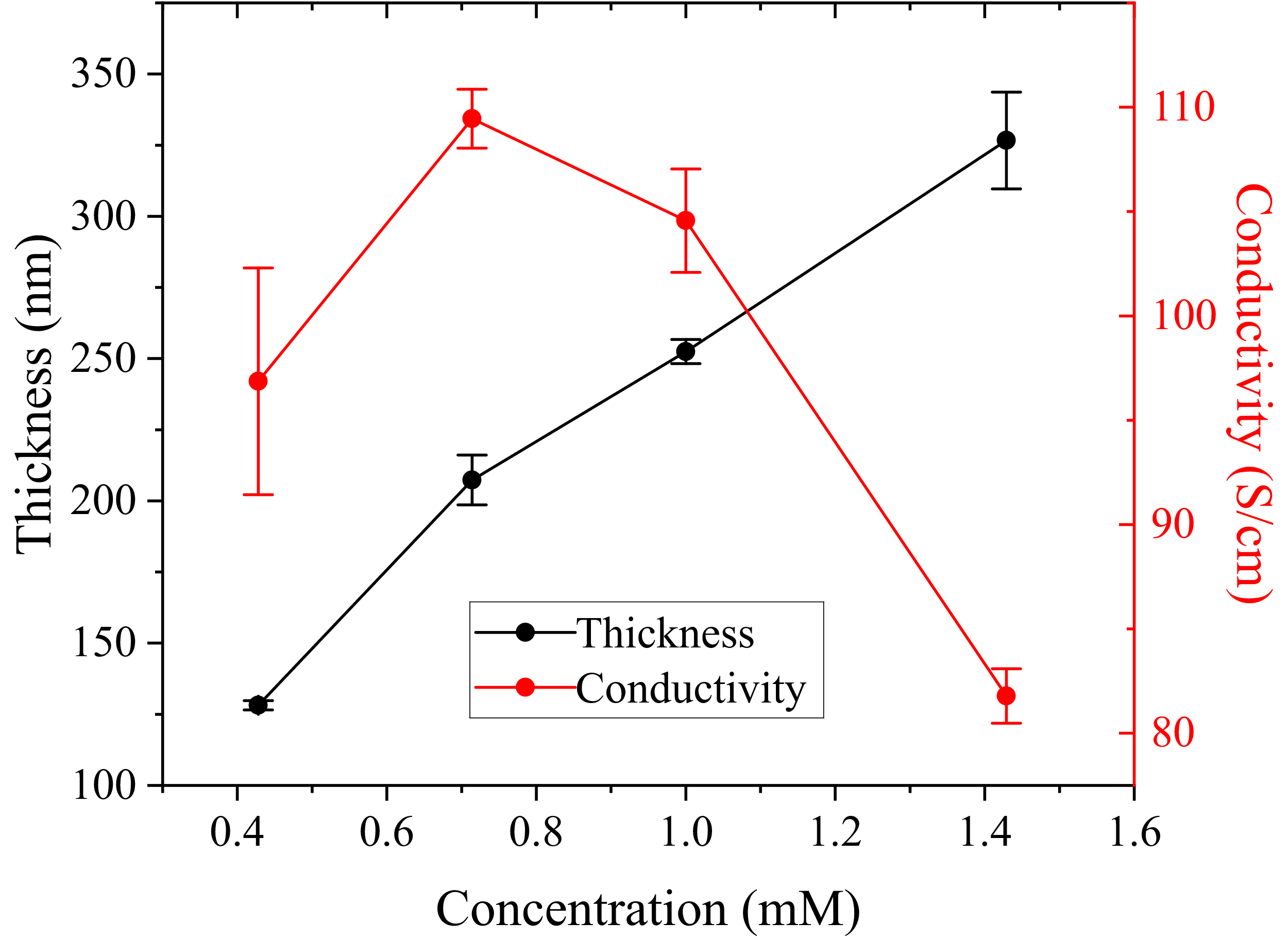


**Figure S7** Thickness and Conductivities of Ni-BHT films synthesized with different concentrations. The ratio of precursor solution is kept to 1 : 1. The data of each reaction time is calculated from three different devices.

In summary, we have optimized the synthesis process of Ni-BHT by exploring precursor ratios, reaction time and concentrations. The optimal condition is a precursor ratio of 0.5 : 0.5 with a reaction time of 2 h.

**Section 2 Morphology and Structure**


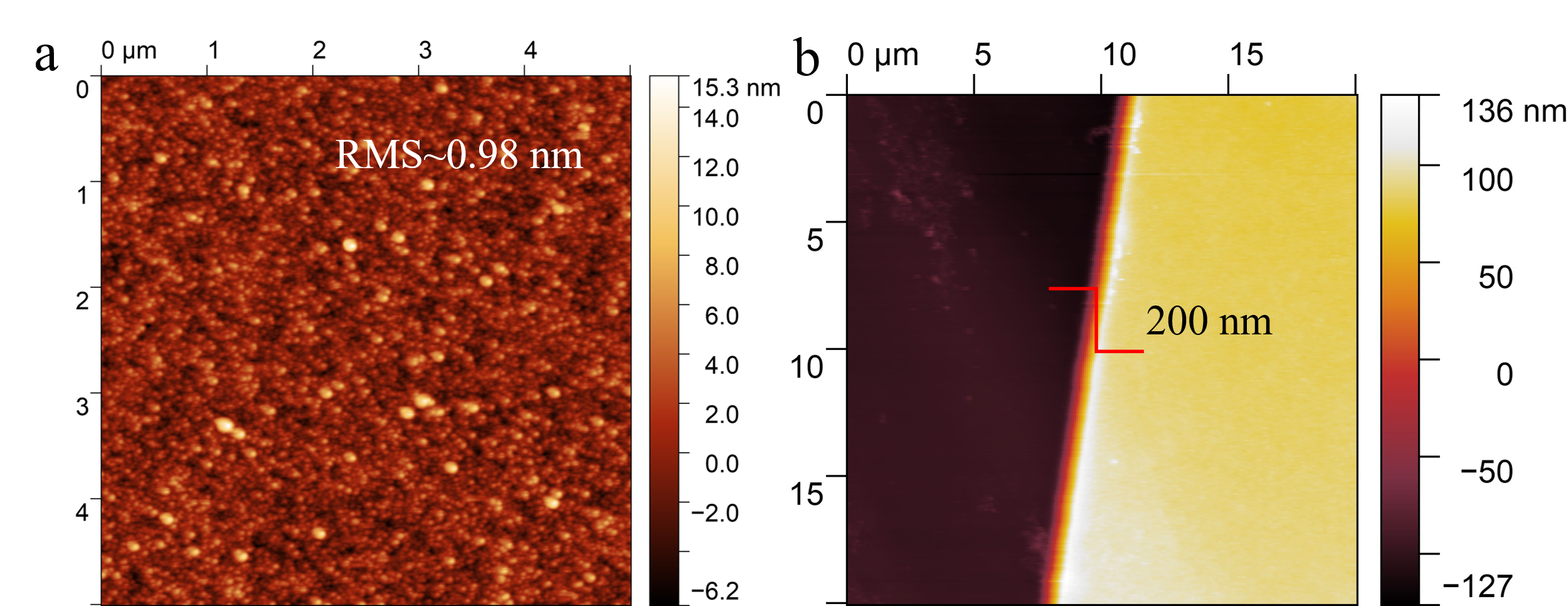


**Figure S8** AFM scan of a. 5 μm*5 μm Ni-BHT surface; b. Boundary of Ni-BHT and glass.


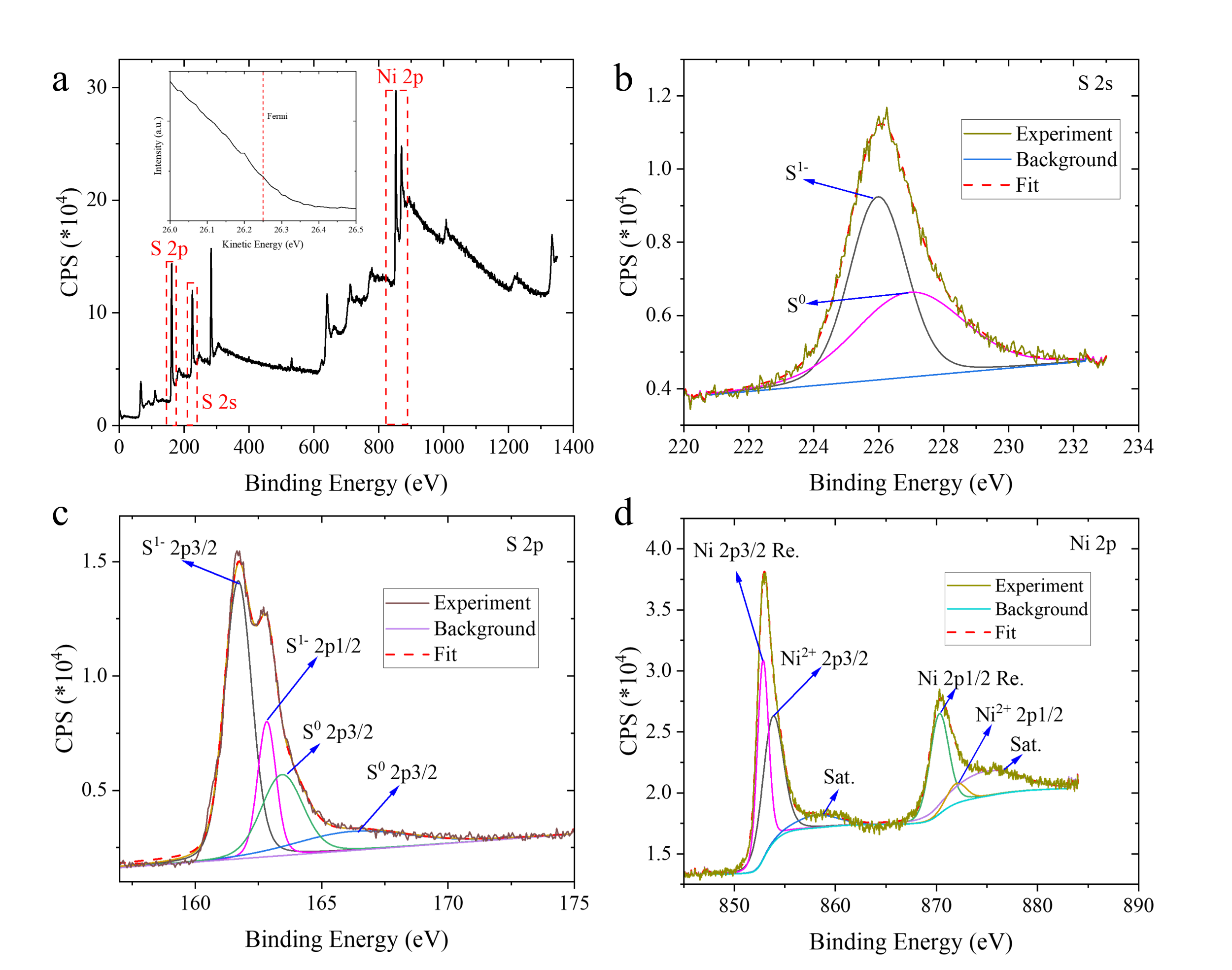


**Figure S9** XPS of Ni-BHT deposited on Si: a. Full spectrum, inset: UPS of the same sample; Deconvolution of XPS high-resolution scan of b. S 2s peak; c. S 2p peak; d. Ni 2p peak. Here, Re. is shorted for “reduced” while Sat. is shorted for “satellite”.


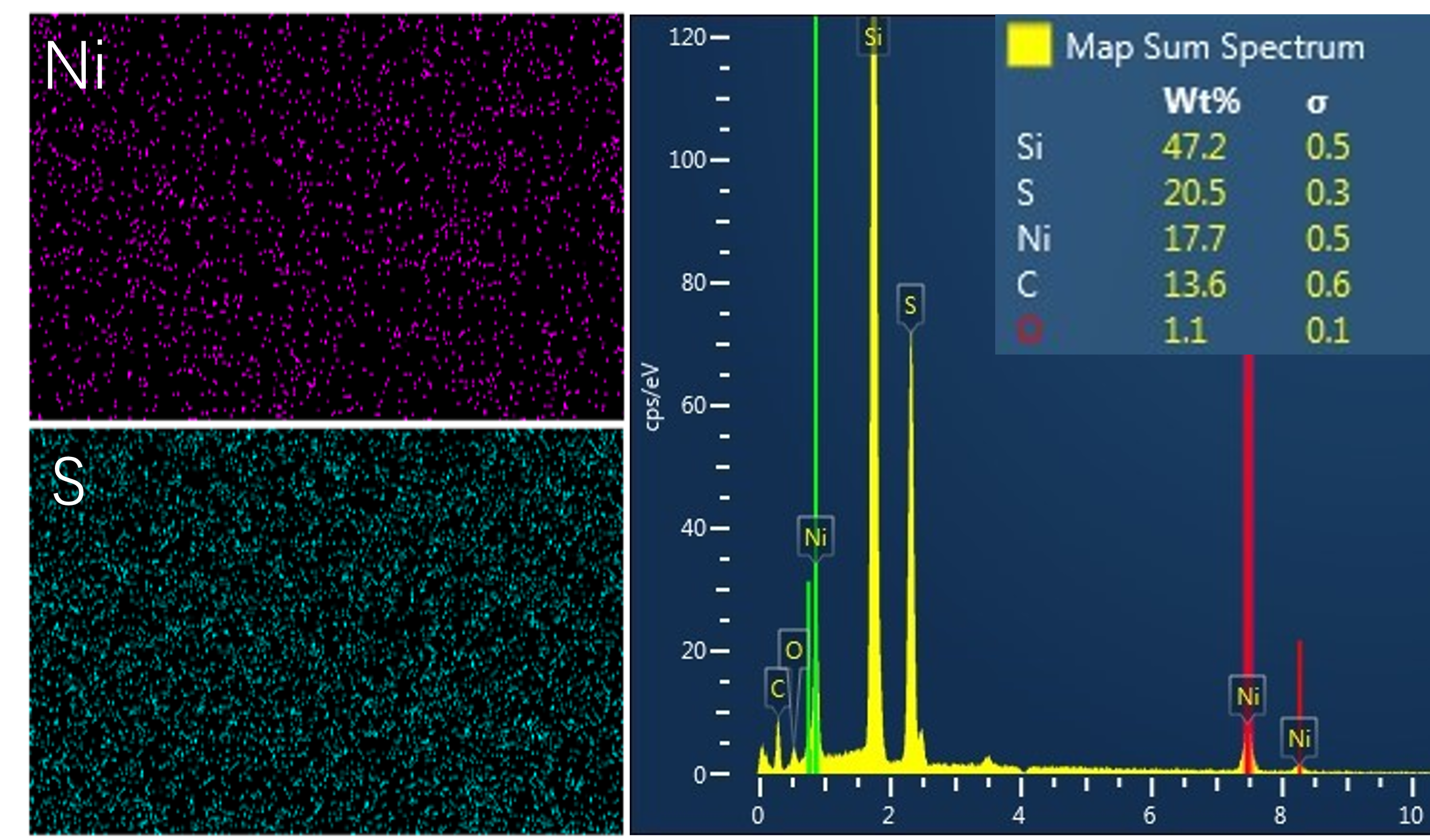


**Figure S10** EDS of Ni-BHT deposited on SiO_2_. Left: Element distribution of Ni and S; Right: Weight percentage of EDS mapping.

**Section 3. Magnetotransport**

Angular MR measurement: MR were measured at 0, 60 and 90 degrees. The angle refers to that of magnetic field to the sample surface. The whole measurement was also based on PPMS where magnetic field varies from -14 T to 14 T.

Extraction of Coulomb gap: The extraction of Coulomb gap is based on the following equation:^[2]^

| $\Delta c={(1-\frac{\varepsilon_{0}}{k})}^{\frac{1}{2}}\frac{k_{B}T_{ES}}{2.8{(4\pi)}^{\frac{1}{2}}}$ | S1 |
| --- | --- |

Where *∆c* is the coulomb gap, *ε_0_* is vacuum permittivity, *k* is dielectric constant, *T_ES_* is defined as the *T_0_* from **equation 1** under ES-VRH model, *k_B_* is Boltzmann constant. *k* and *T_ES_* can be related as:^[2]^

| $T_{ES}=\frac{2.8e^{2}}{kk_{B}L_{c}}$ | S2 |
| --- | --- |

Substitute *T_ES_*, *L_c_*, ε_0_ values into the above equations, *∆c* can be extracted.


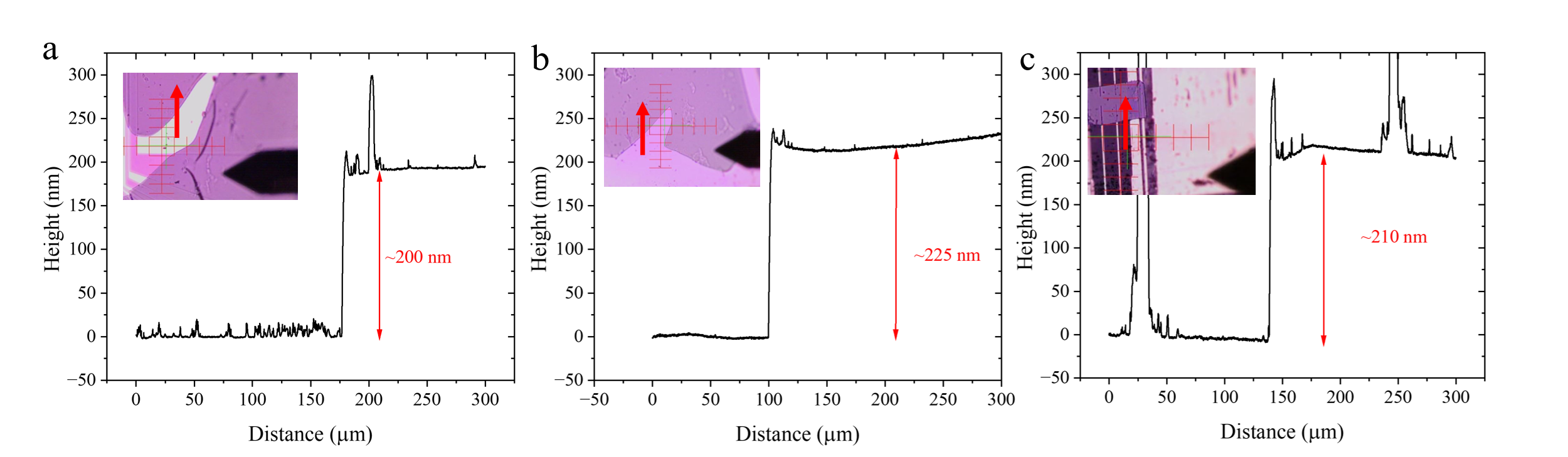


**Figure S11** Dektak profiles of 300-μm linecut scans of Ni-BHT films on top of a. gold; b. silicon oxide; c. device channel. The red arrows indicate the scanning path of probe.


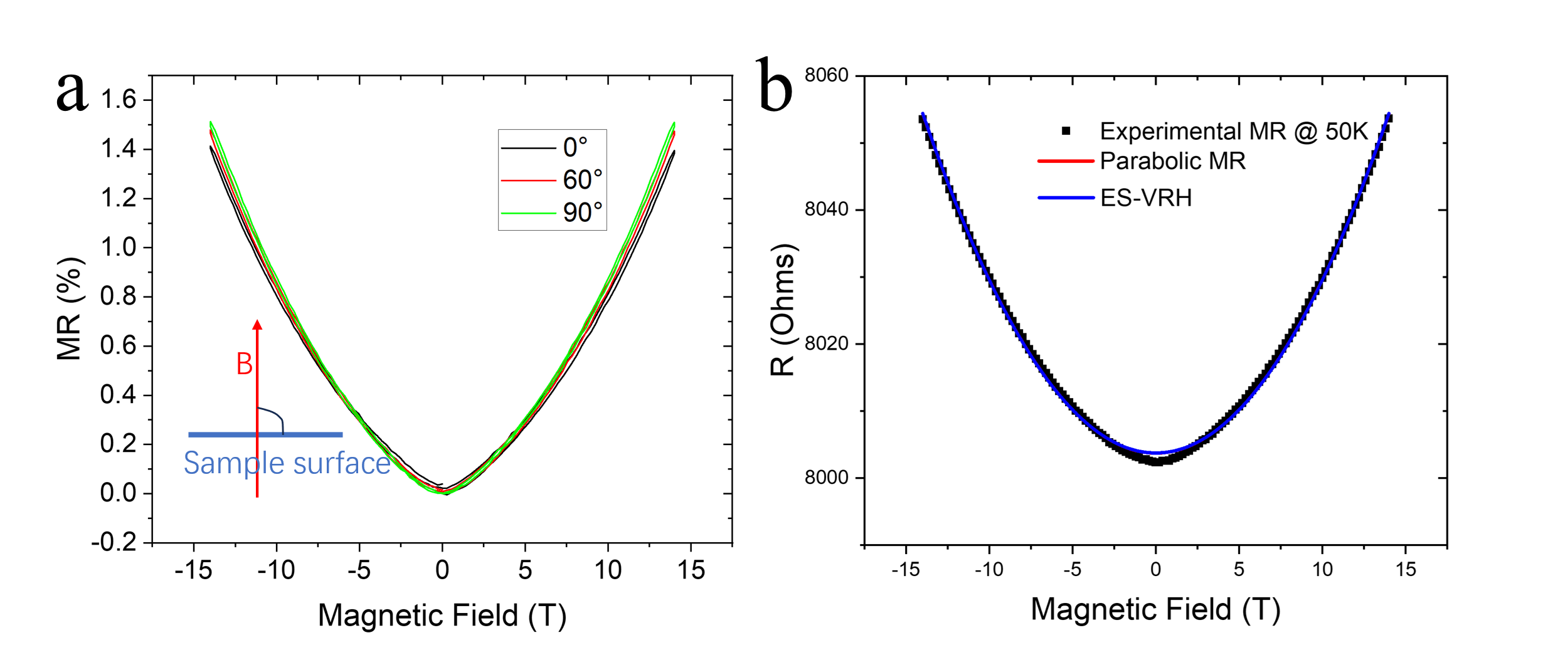


**Figure S12** a. Angular dependent MR measurement of Ni-BHT at 30 K; b. MR at 50K and the fitting curve according to parabolic and ES-VRH fitting methods.


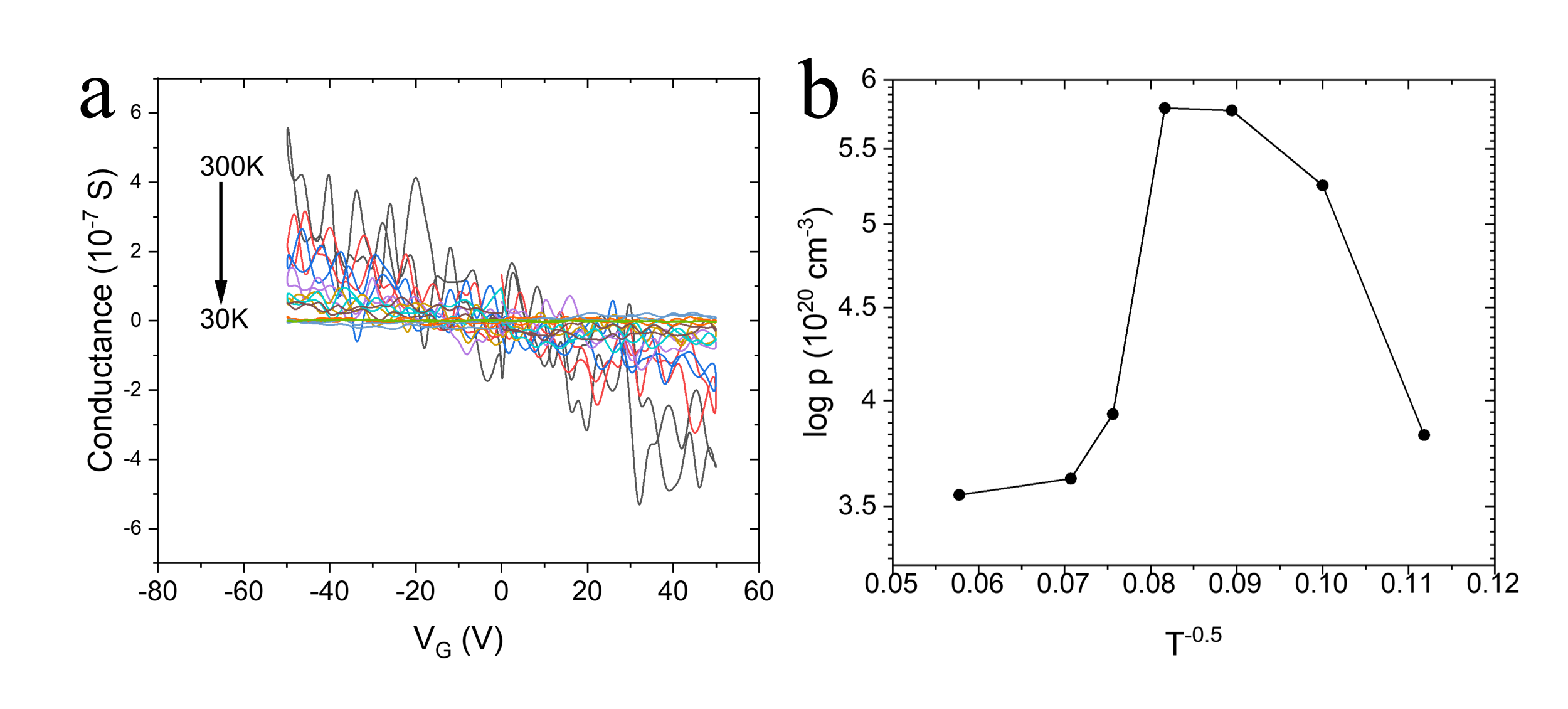


**Figure S13** a. Transfer curves of Ni-BHT measured from V_G_= -50 V to 50 V (the conductance at zero gate voltage is subtracted to better compare the transfer curves at different temperatures); b. Extracted temperature dependent carrier densities (*p*) from FET transfer curves. The noisy transfer curve might be due to the rough interface between Ni-BHT and SiO_2_.

| **Table S1** Electrical performance of three different devices at room temperature (~295 K) (D1/2/3 refers to device1/2/3, SD stands for standard deviation) | | | |
| --- | --- | --- | --- |
| Devices | Dimension (*l*, *w, t*) [μm] | Conductivity  [S cm^-1^] | Mobility  [cm^2^ V^-1^ s^-1^] |
| D1 | 420 *310 *0.21 | 107.6 | 2.21 |
| D2 | 420 *310 *0.21 | 100.6 | 1.53 |
| D3 | 420 *310 *0.19 | 108.7 | 1.74 |
| Average | N/A | 105.63 | 1.82 |
| SD |  | 3.59 | 0.28 |


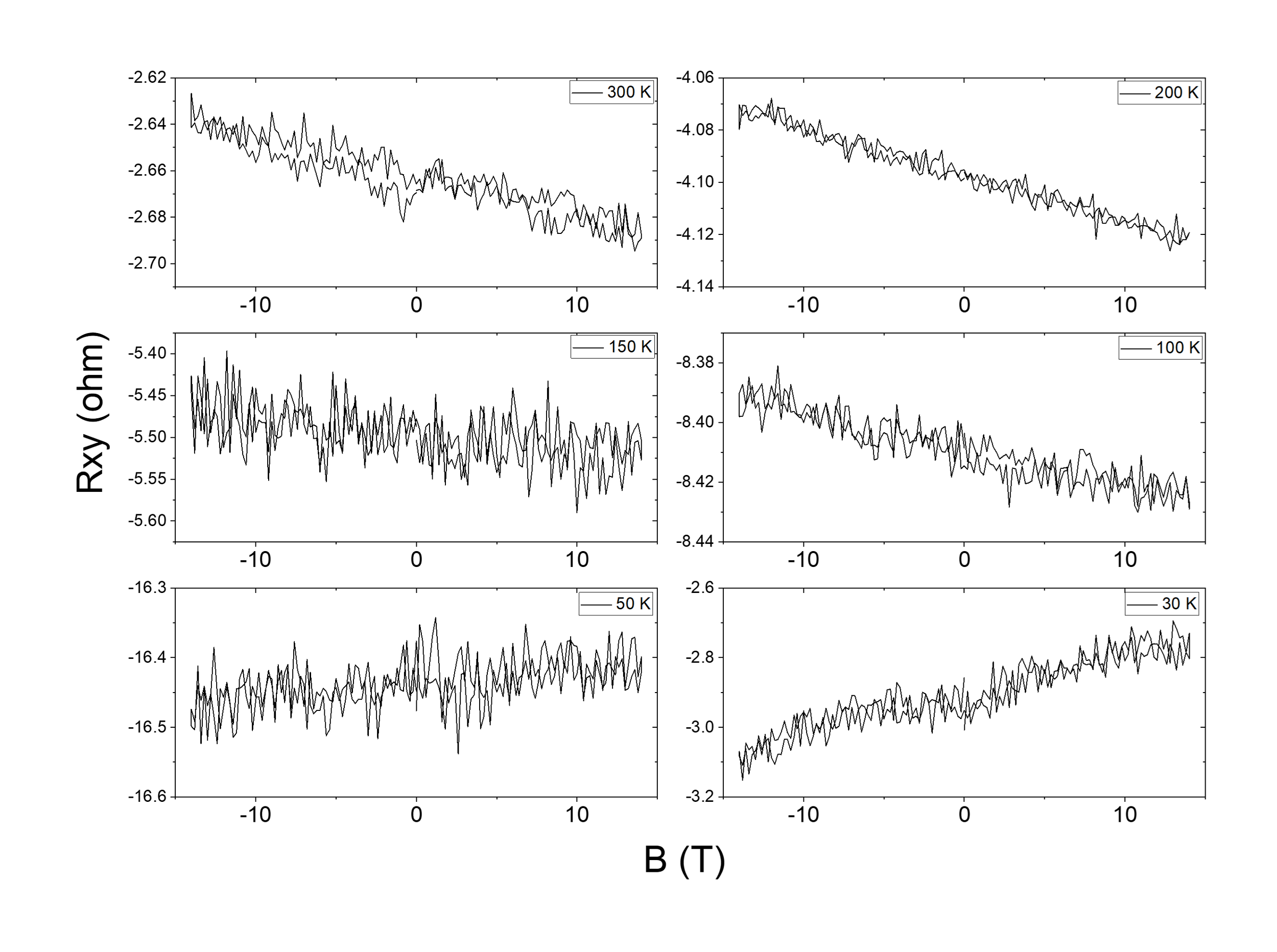


**Figure S14** Temperature dependent Hall effect of Ni-BHT.


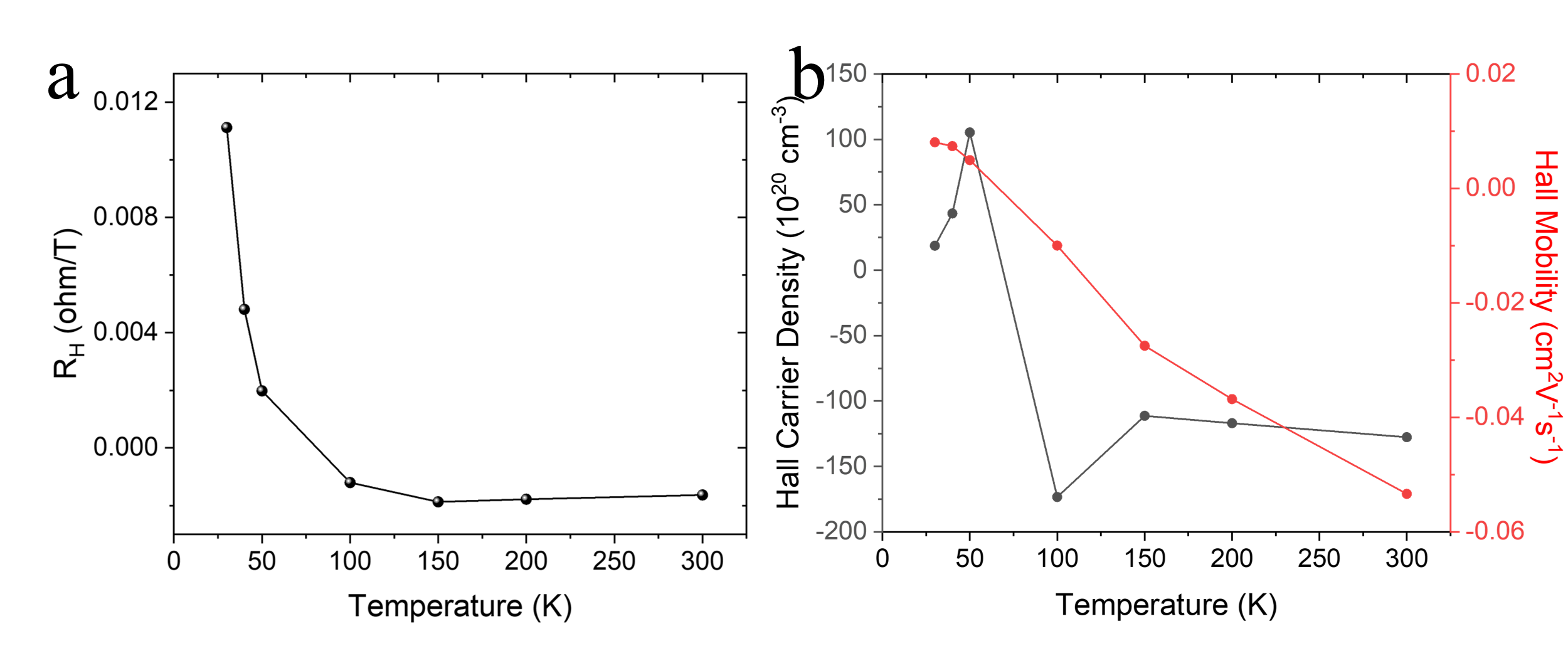


**Figure S15** a. Hall coefficients extracted at different temperatures; b. Calculated Hall carrier density and Hall mobility at different temperatures. A simple single carrier model is used for those calculations.

**3.1 Hall anomaly in Ni-BHT**

Hall effect measurements of Ni-BHT are shown in **Figure S14**. N-type dominant transport at high temperatures is revealed from the negative sign of the Hall coefficient (*R_H_*). When the temperature decreases to near 50 K, *R_H_* becomes positive and increases strongly at lower temperature (**Figure S15a**). Based on a single carrier model, the Hall carrier densities and mobilities at each temperature can be extracted as shown in **Figure S15b**. However, these numbers are unlikely to be reliable as the carrier density reaches very high values of 10^22^ cm^-3^, which would correspond to around 2.83 carriers per unit cell at 300 K (assuming a perfect crystal, volume of unit cell = 221.64 Å^3^). One explanation for the temperature dependence of the polarity of the Hall voltage could be, that Ni-BHT is in fact ambipolar, i.e. both electron and hole bands within the band structure contribute to conduction, similar with Cu-BHT as reported elsewhere.^[1,3,4]^ However, there could be alternative reasons for the small Hall voltages measured in Ni-BHT. The most likely explanation of the discrepancy between Hall effect and FET measurements could be the sign anomalies that are expected in the Hall effect for strongly disordered systems, which is widely observed in amorphous materials.^[5–7]^ In hopping transport, localized charge carriers cannot be given a defined drift velocity leading to an ineffective Hall effect.^[8]^ The Hall effect observed in hopping transport can be understood as self-interference effect of the electron wave function which propagates along different hopping paths in the magnetic field. In this scenario, sign of *R_H_* is not only dependent on the polarity of carriers:

| $Sign R_{H}=Sign\left[ \epsilon^{n+1}\prod_{i=1}^{n} J_{i,i+1} \right]$ | S3 |
| --- | --- |

Here, 𝜖=±1 according to the polarity of carriers (hole or electron), *n* is the number of sites that are involved in a hopping event.^[9]^ For an odd number of *n*, sign of *R_H_* is dependent on the transfer integrals around the closed path ($\prod_{i=1}^{n} J_{i,i+1}$), which can be positive or negative. An example is shown in **Figure S16**. When a three-site geometry is involved (**Figure S16a, b,** n=3), 𝜖^n+1^ is positive while $\prod_{i=1}^{3} J_{i,i+1}$ is positive for antibonding and negative for bonding orbitals. This would lead to a positive *R_H_* sign for electrons transport through antibonding orbitals and negative sign for holes transport through bonding orbitals. On the contrary, $\prod_{i=1}^{4} J_{i,i+1}$ is positive for both antibonding and bonding orbitals in a four-site geometry (**Figure S16c, d,** n=4). Therefore, *R_H_* sign is determined by the polarity of carriers, that is, negative for electron and positive for holes. Unfortunately, in such a hopping transport regime in which the Hall effect is governed by interference effects in multi-site hopping it is very difficult to deduce quantitative insights into the transport physics from analysis of the Hall effect. More details can be found in reference^[9]^.


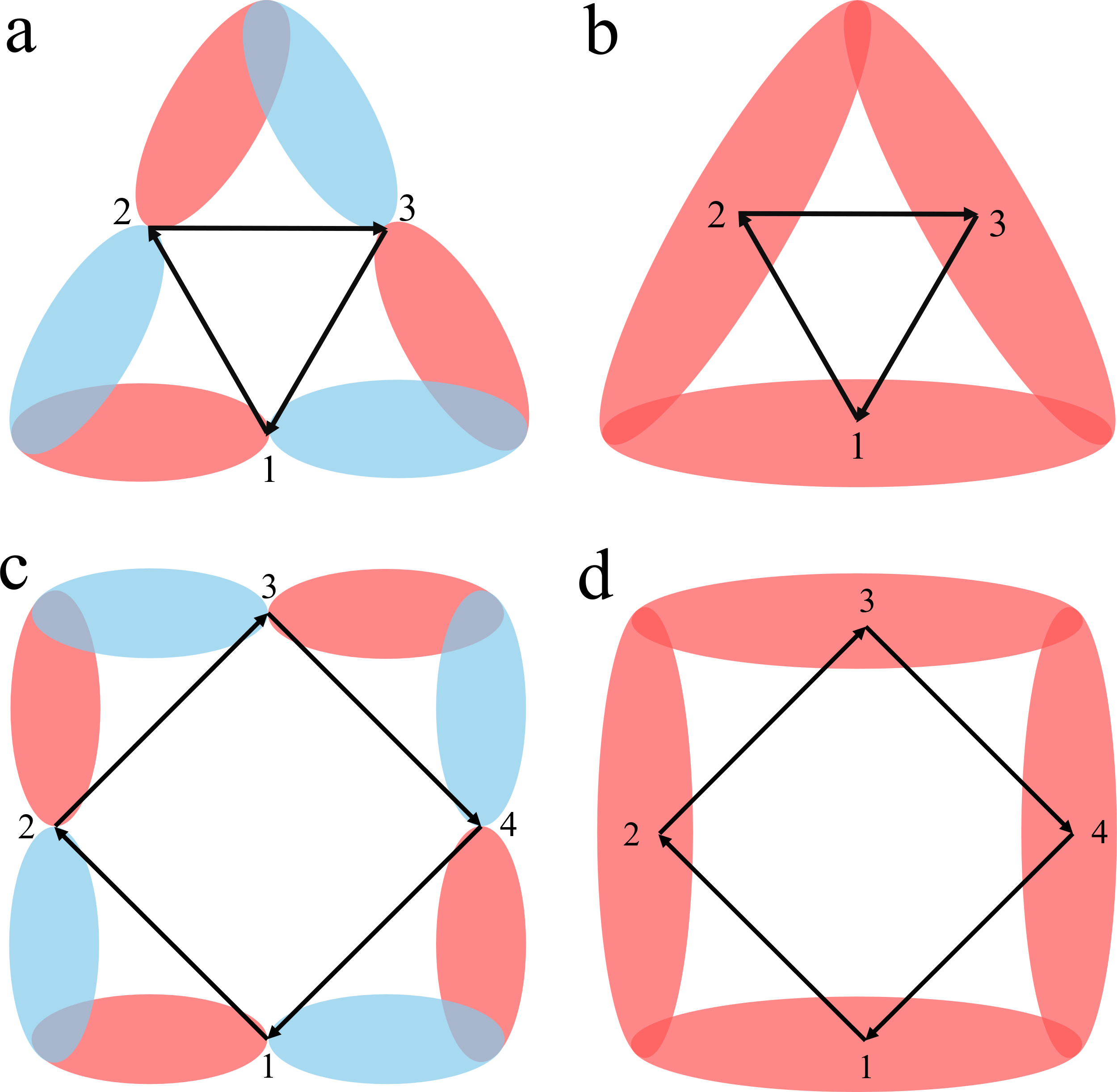


**Figure S16** Schematic of closed-loop processes involving antibonding (a, c) and bonding (b, d) orbitals in three- (a, b) and four-site (c, d) structures. Arrows indicate the movement of carriers between the centroids of the orbitals, with numbered ‘sites’ marking the locations of carrier transitions. The chosen phases of the antibonding orbitals ensure that each transfer integral is positive while that of bonding orbitals forms a negative transfer integral.

**3.2 FET vs Hall effect measurement**

Hall effect measurements probe the bulk (or averaged) transport properties of a film by detecting the voltage generated when mobile charge carriers experience a Lorentz force under an applied magnetic field. This method is effective in materials with band-like transport, where carriers have well-defined velocities. However, in systems dominated by hopping transport, such as Ni-BHT, the Hall effect becomes complicated because carriers "hop" between localized sites and do not possess a continuous velocity, meaning they do not effectively experience the Lorentz force.^[10]^ As a result, the Hall signal can be suppressed or even show sign anomalies (see Section 3.1). Additionally, even in band transport systems, low carrier mobility can lead to a poor signal-to-noise ratio and unreliable results.^[11]^ Therefore, Hall measurements are strongly influenced by both the transport mechanism and the intrinsic mobility of the material.

In contrast, FET measurements are primarily sensitive to the modulation of carrier density and transport behavior at the interface between the semiconductor film and the gate dielectric. While very high intrinsic carrier density (such as metals) can reduce the degree of field-effect modulation and make the signal harder to detect, the method is less affected by whether transport occurs via band conduction or hopping, as it only relies on the modulation of carrier density. However, interface quality also plays a crucial role: gaps or poor contact at the film/dielectric interface can reduce effective charge accumulation, leading to underestimation of mobility.^[12]^ Thus, while the FET mobility is affected by interfacial and structural factors, it remains a more reliable probe of transport character in disordered or hopping systems like Ni-BHT.

**Section 4 Electrolyte Gating**


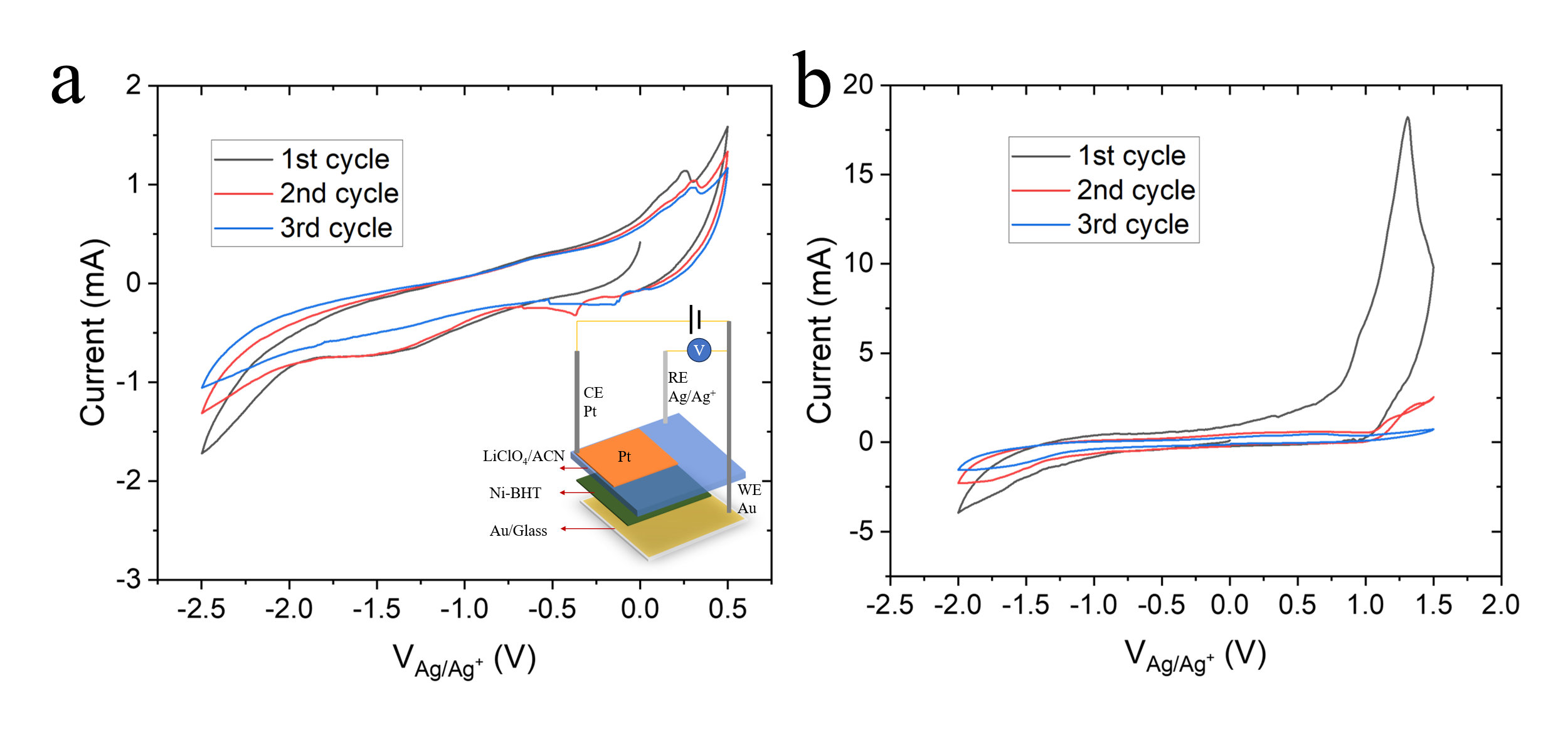


**Figure S17** CV measurements of Ni-BHT deposited on gold (inset in a). a. -2.5 to 0.5 V; b. -2.0 V to 1.5 V.

**CV measurement:** Ni-BHT film was deposited on top of Au (20 nm, evaporated onto glass with 3nm Cr as adhesion layer). LiClO_4_/ACN (0.2 M) was used as electrolyte. CV was measured using a portable probe station connected to a potentiostat (PalmSens4).


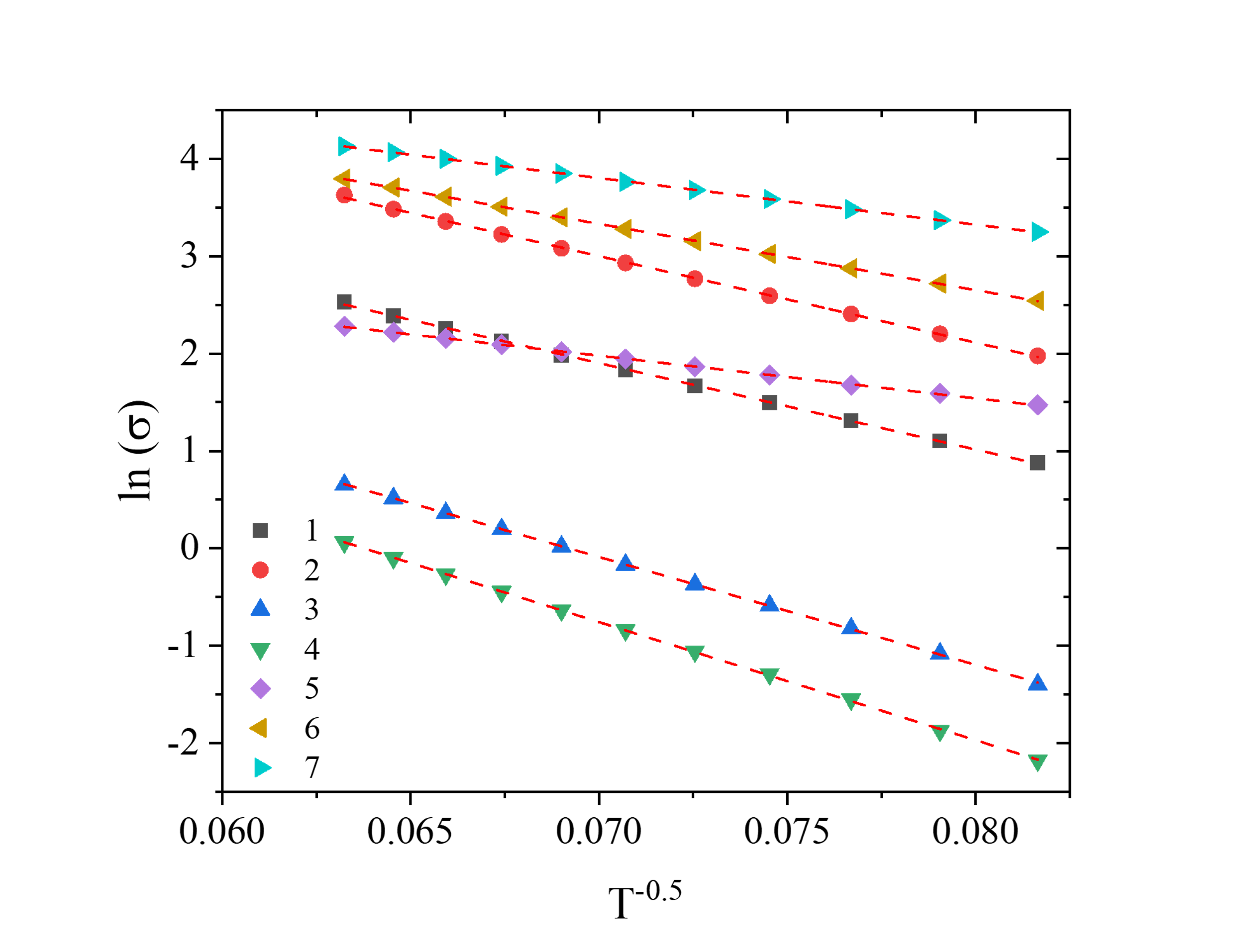


**Figure S18** ln (*𝜎*) plotted against *T*^-0.5^ for different doping states.

| **Table S2** Thermoelectric performances of three different devices at 250 K (D1/2/3 refers to device1/2/3; dimension (length: *l*, width: *w*, thickness: *t*, all in unit of μm), Conductivity (*𝜎*, unit: S cm^-1^), Seebeck Coefficient (*S*, unit: μV K^-1^), power factor (*PF*, unit: μW m^-1^K^-2^); Ave. stands for average; SD stands for standard deviation) | | | | | | | | | |
| --- | --- | --- | --- | --- | --- | --- | --- | --- | --- |
| States | State #0 | | | State #1 | | | State #2 | | |
| Parameters | *𝜎* | *S* | *PF* | *𝜎* | *S* | *PF* | *𝜎* | *S* | *PF* |
| **D1**  *l*: 420  *w*: 150  *t*: 0.21 | 92.49 | 14.40 | 1.92 | 9.60 | -38.16 | 1.40 | 39.50 | -44.50 | 7.82 |
| **D2**  *l*: 420  *w*: 180  *t*: 0.21 | 100.51 | 13.40 | 1.80 | 8.69 | -36.04 | 1.13 | 36.88 | -41.99 | 6.51 |
| **D3**  *l*: 420  *w*: 140  *t*: 0.21 | 89.08 | 14.51 | 1.87 | 12.61 | -36.50 | 1.68 | 37.72 | -43.05 | 6.99 |
| Ave. | 94.03 | 14.10 | 1.86 | 10.30 | -36.90 | 1.40 | 38.03 | -43.18 | 7.11 |
| SD | 4.79 | 0.50 | 0.05 | 1.67 | 0.94 | 0.23 | 1.09 | 1.03 | 0.54 |


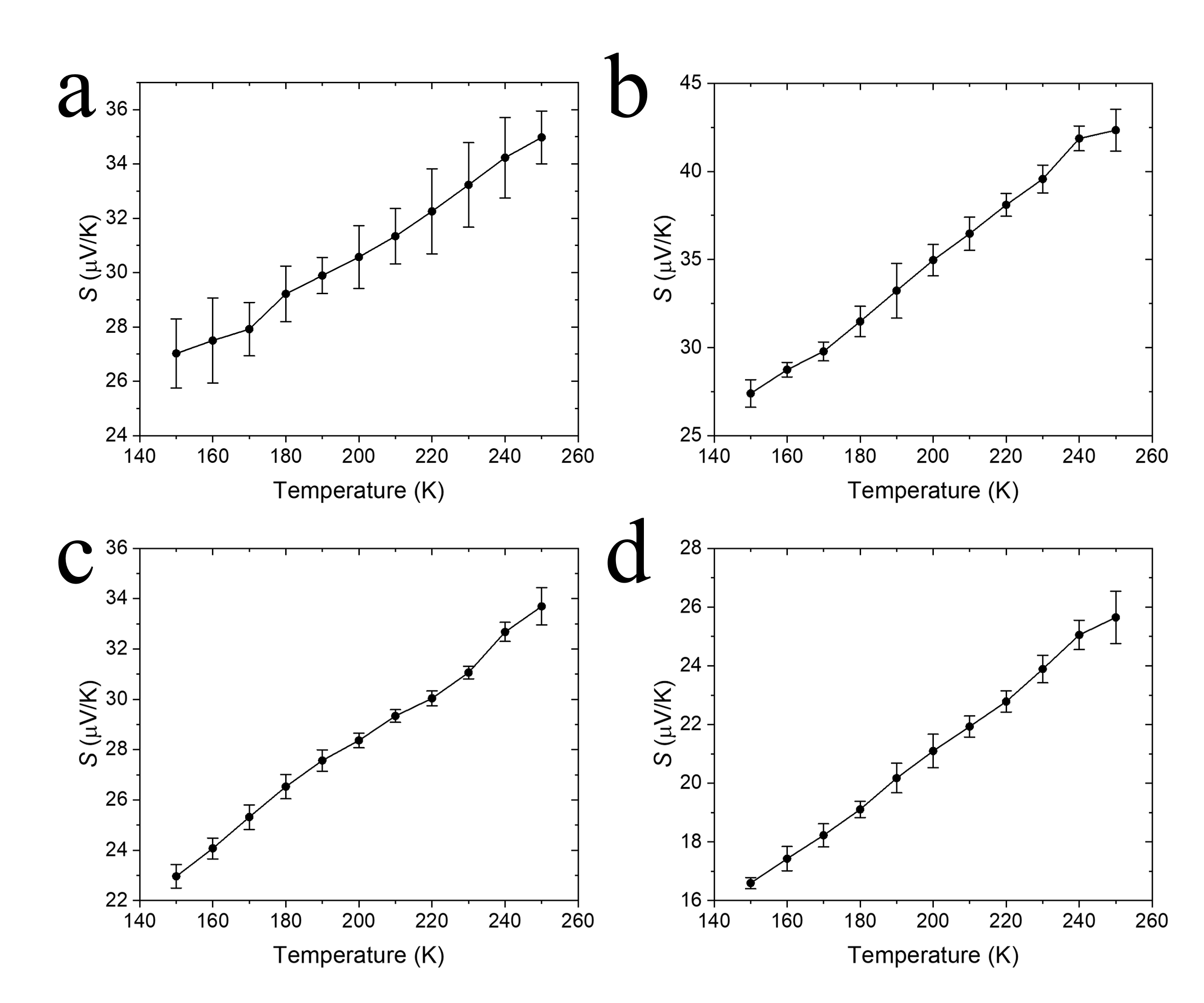


**Figure S19** Temperature dependent Seebeck coefficients of a. state 4; b. state 5; c. state 6; d. state 7.


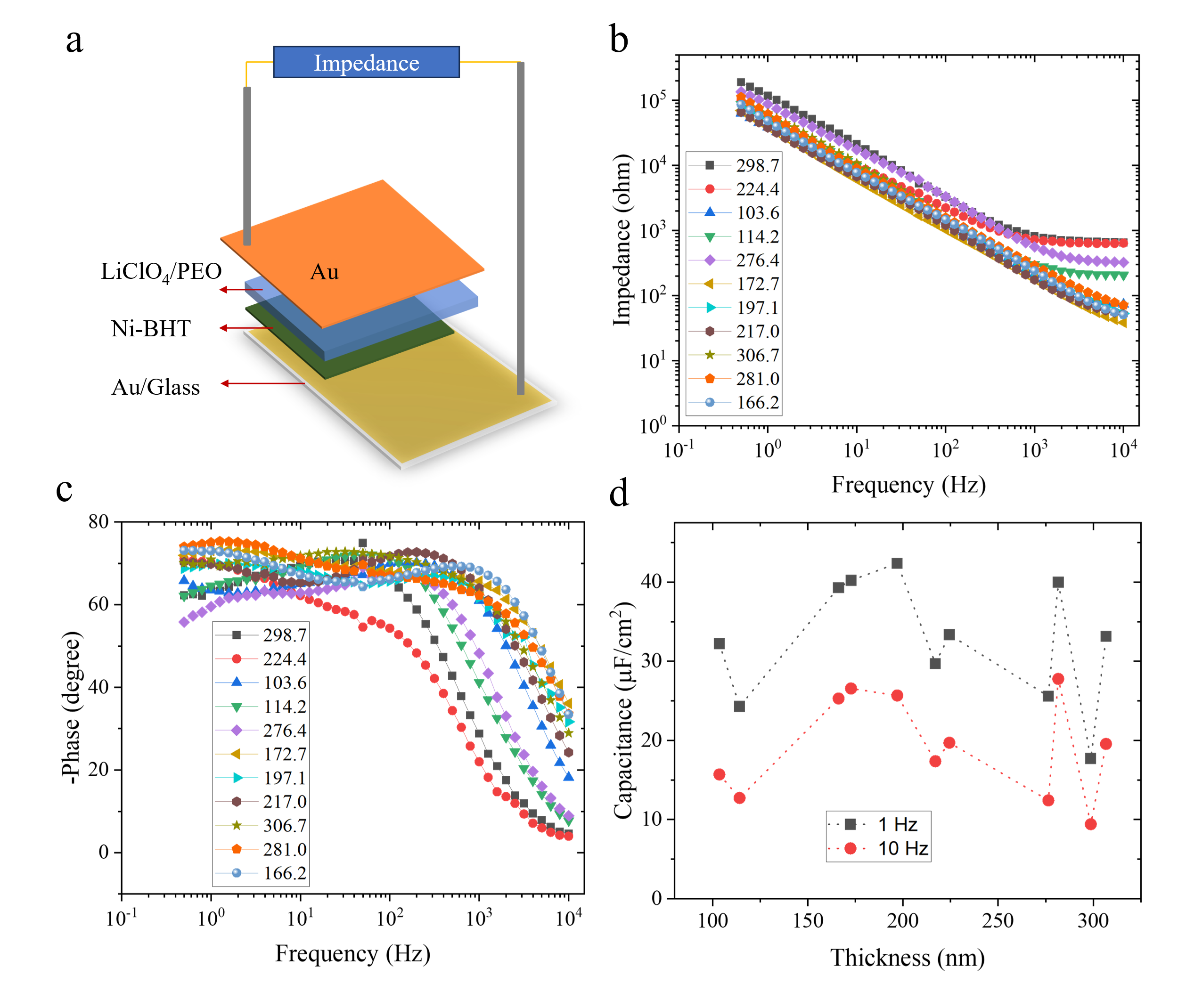


**Figure S20** a. Impedance measurement of the fabricated simple capacitor with a structure of Au/LiClO4(PEO)/Ni-BHT/Au; b. Frequency dependant impedance of Ni-BHT films with different thickness; c. Frequency dependant phase of Ni-BHT films with different thickness; d. Thickness dependent capacitance of Ni-BHT films with different thickness.

**Impedance Measurement:** The impedance measurement was based on a simple capacitor. First, Ni-BHT film was transferred onto glass substrate with 25 nm Au deposited on the surface (Au/glass). LiClO_4_/PEO electrolyte films were drop-casted from solution on top of both the Ni-BHT and a bare Au/glass substrate. The Au electrode on glass have a much larger area than the Ni-BHT film. After fully drying of the solution, the two substates were put together (structure shown in **Figure S20a**) and connected to a PlamSens4 potentiostat for impedance measurements. The measurements were taken from 10000 Hz to 0.5 Hz with an AC voltage of 0.01 V and a DC voltage of 0 V. We observe no correlation between capacitance and thickness, which suggests that the gating involves an interfacial double formation process. There are significant sample-to-sample variations in the measured capacitance, which could be explained with variations in the roughness of the films, which will affect the magnitude of the double layer capacitance.


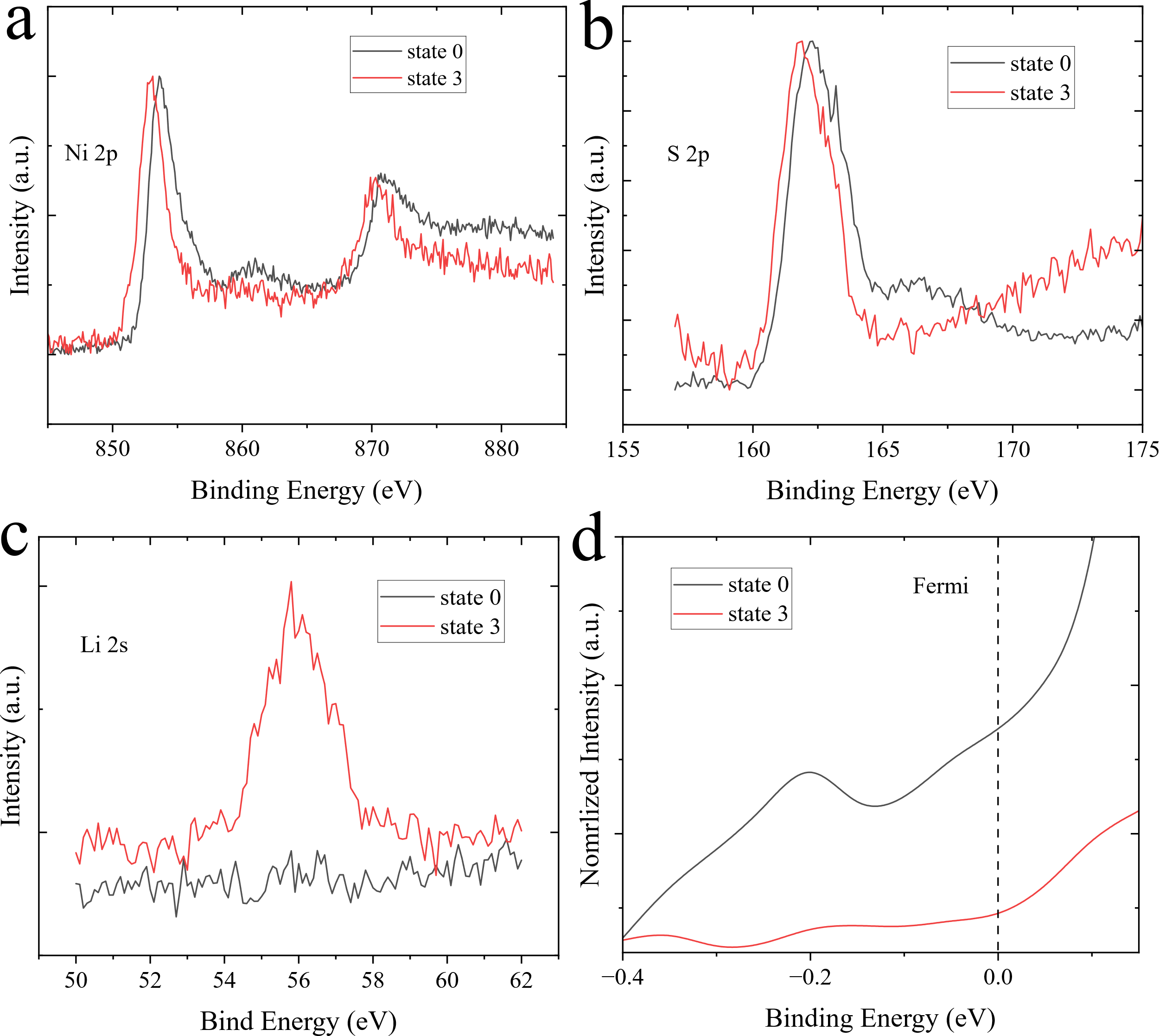


**Figure S21 Photoelectron spectroscopy of Ni-BHT film at state 0 and state 3.** XPS of a. Ni 2p; b. S 2p; c. Li 2s; d. UPS of Ni-BTH film at state 0 and 3. A depletion of states at the Fermi level of Ni-BHT has been observed from UPS, which may be due to the structural changes of Ni-BHT and electron injection into the film which shifts the Fermi level.

**XPS sample preparation of Ni-BHT film prepared at state 3:** Ni-BHT film was gated to state 3 at 60 ℃ with gating voltage of 3V. The temperature was decreased to 10 ℃ while the gating voltage is still applied. The gating voltage was removed when the temperature stabilized at 10 ℃. Then, the sample was quickly transferred to a glovebox using a N_2_ filled transfer tube. The sample was placed on a spin coater while the speed is set to be 5000 rpm with an acceleration of 1000 rpm/s. A drop of ACN was dropped on top of the sample. A thin layer of organic film (PEO) will form at the surface of the sample. After spin coating, the sample was place on the XPS holder and put in a N_2_ filled transfer tube, avoiding contact with air.

**XPS measurement:** The transfer tube was inserted into XPS sample-loading chamber. The sample was loaded using a mechanical arm and moved to XPS chamber. Before XPS/UPS measurement, we need to remove the thin organic layer on top of the sample. An Argon asher was used for etching the surface. The etching thickness is around 100 nm which is enough to remove all the organic layer. The whole process was in high vacuum (<10^-6^ mbar) to avoid air contact. After the etching process, XPS and UPS spectra were taken for the gated sample.


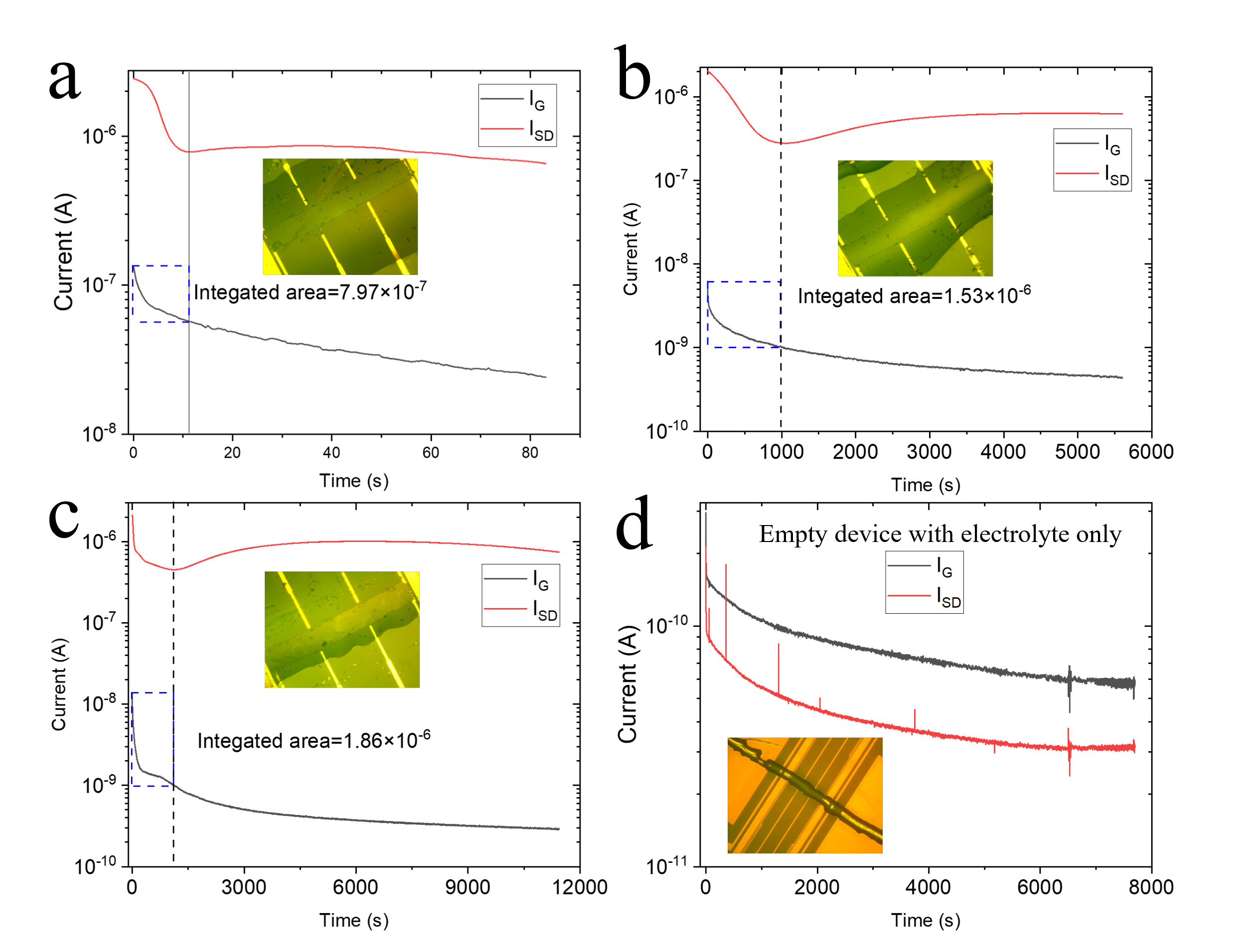


**Figure S22** a-c: *I_G_/I_SD_* *vs* time plots of three different samples (insets are the optical images of each sample); d. *I_G_/I_SD_ vs* time plots of an empty device with only electrolyte and Au electrodes, exhibiting much smaller gate current.

| **Table S3** Integrated charges and calculated Li^+^ density from **Figure S22 a-c** | | | |
| --- | --- | --- | --- |
|  | 1 | 2 | 3 |
| *N* | 4.98*10^12^ | 9.55*10^12^ | 1.16*10^13^ |
| *V* [cm^3^] | 3.36*10^-8^ | 4.20*10^-8^ | 4.20*10^-8^ |
| *n* [cm^-3^] | 1.48*10^21^ | 2.27*10^21^ | 2.77*10^21^ |

**Reference**

[1] X. Huang, P. Sheng, Z. Tu, F. Zhang, J. Wang, H. Geng, Y. Zou, C. Di, Y. Yi, Y. Sun, W. Xu, D. Zhu, *Nat. Commun.* **2015**, *6*, 7408.

[2] S. Wang, M. Ha, M. Manno, C. Daniel Frisbie, C. Leighton, *Nat. Commun.* **2012**, *3*, 1210.

[3] R. Tsuchikawa, N. Lotfizadeh, N. Lahiri, S. Liu, M. Lach, C. Slam, J. Louie, V. V. Deshpande, *Phys. status solidi* **2020**, *217*, 2000437.

[4] T. Wu, X. Ren, D. Cornil, C. Quarti, I. E. Jacobs, L. Zhang, N. Fukui, D. Beljonne, H. Nishihara, H. Sirringhaus, *Sci. Adv.* **2025**, *11*, eadt9196.

[5] M. Grunewald, P. Thomas, D. Wurtz, *J. Phys. C Solid State Phys.* **1981**, *14*, 4083.

[6] C. E. Nebel, M. Rother, M. Stutzmann, C. Summonte, M. Heintze, *Philos. Mag. Lett.* **1996**, *74*, 455.

[7] M. Jaime, H. T. Hardner, M. B. Salamon, M. Rubinstein, P. Dorsey, D. Emin, *Phys. Rev. Lett.* **1997**, *78*, 951.

[8] A. Avdonin, P. Skupiński, K. Grasza, *Phys. B Condens. Matter* **2016**, *483*, 13.

[9] D. Emin, *Philos. Mag. A J. Theor. Exp. Appl. Phys.* **1977**, *35*, 1189.

[10] H. T. Yi, Y. N. Gartstein, V. Podzorov, *Sci. Rep.* **2016**, *6*, 23650.

[11] F. Werner, *J. Appl. Phys.* **2017**, *122*, 135306.

[12] X. Sun, C. Di, Y. Liu, *J. Mater. Chem.* **2010**, *20*, 2599.
